# NADPH Oxidase-Related Oxidative Stress in Parkinson’s Disease-Associated Cognitive Impairment: An Evidence-Focused Review

**DOI:** 10.3390/antiox15101367

**Published:** 2026-10-21

**Authors:** Dong-Hee Choi, In-Ae Choi

**Affiliations:** 1 Department of Medical Science, School of Medicine, Konkuk University, Chungju-si 27478, Chungcheongbuk-do, Republic of Korea; 2 Department of Occupational Therapy, Division of Health, Baekseok University, Cheonan-si 31065, Chung-cheongnam-do, Republic of Korea; inaes2@bu.ac.kr

**Keywords:** Parkinson’s disease, cognitive impairment, Parkinson’s disease dementia, NADPH oxidase, oxidative stress, NOX4, NOX2/gp91phox, microglia, neuroinflammation

## Abstract

Cognitive impairment is a major non-motor manifestation of Parkinson’s disease (PD); however, evidence linking specific NADPH oxidase (NOX) isoforms to cognition-related outcomes remains incompletely integrated. In this evidence-focused narrative review, a structured four-database search identified 12 studies for study-level synthesis: 10 rodent studies involving cognitive assessment in PD-relevant models and 2 contextual studies. The 10 core studies were appraised according to the directness with which NOX involvement was examined in relation to cognitive outcomes. The most direct experimental support centered on hippocampal NOX4- and NOX2/gp91phox-associated microglial signaling. Dentate-gyrus-targeted NOX4 knockdown attenuated learning and memory deficits, with concurrent reductions in proteinopathy-related changes. CD68-driven gp91phox knockdown similarly attenuated these deficits alongside reductions in inflammatory responses and neuronal and synaptic injury. Apocynin was classified as a NOX-targeted pharmacological intervention and improved learning and memory performance. However, its lack of target and isoform specificity precluded definitive attribution of the behavioral effect to NOX inhibition. Other studies contributed partial or supportive evidence, while complementary pathway-level evidence linked cognition-related inflammatory signaling to NOX1/NOX2 dependence. Human postmortem and canine biomarker studies have provided clinicopathological and comparative peripheral biomarker context, respectively, but they have not established a NOX–cognition relationship in human PD. Overall, the evidence suggests that NOX-related oxidative stress contributes to the development of learning and memory deficits in PD-relevant models. Because the principal NOX4 and gp91phox genetic interventions preceded model induction, the observed effects are best interpreted as protection during deficit development rather than reversal of established cognitive impairment. Cross-model validation, NOX-directed interventions initiated after cognitive impairment has been confirmed, and studies in cognitively characterized human PD cohorts are needed to establish translational relevance.

## 1. Introduction

Cognitive impairment is one of the most clinically consequential non-motor manifestations of Parkinson’s disease (PD), with substantial effects on functional independence, quality of life, prognosis, and caregiver burden. In PD, cognitive impairment spans a clinical spectrum from subtle cognitive changes to Parkinson’s disease mild cognitive impairment (PD-MCI) and Parkinson’s disease dementia (PDD). The Movement Disorder Society Task Force criteria define PD-MCI as objectively supported cognitive decline that does not substantially interfere with functional independence, whereas PDD refers to a dementia syndrome that develops in established PD and impairs daily functioning [1,2]. The course of cognitive impairment is heterogeneous and not uniformly progressive. Within 3 years, approximately 25% of cognitively normal patients with PD were reported to develop PD-MCI, and 2% were reported to develop dementia, whereas 20% of patients with PD-MCI progressed to dementia and 28% reverted to normal cognitive classification [3]. A more recent meta-analysis estimated a pooled PDD incidence of 4.45 per 100 person-years and a 3.25-fold higher risk of dementia in individuals with PD relative to those without it [4]. These observations identify PD-MCI as a clinically important but dynamic risk state, and highlight the need to define the biological processes that influence the emergence and progression of cognitive decline.

PD-associated cognitive impairment is also biologically heterogeneous and extends beyond nigrostriatal dopaminergic degeneration. Its clinical expression may reflect dysfunction across dopaminergic, cholinergic, noradrenergic, and serotonergic systems, together with pathology affecting distributed cortical, limbic, and subcortical networks [5]. In PDD, limbic and neocortical Lewy body and neurite pathologies are among the strongest neuropathological correlates of dementia; however, their burden does not fully account for cognitive status across all patients [6,7]. Alzheimer-type co-pathology involving amyloid-β plaques and tau neurofibrillary tangles occurs in a substantial subset of PDD cases and may interact with α-synuclein pathology, modifying the clinical phenotype and prognosis [6,8]. Consistent with this biological heterogeneity, PD-MCI also shows variable clinical presentations and cognitive profiles [9]. Together, these observations support a multisystem and multi-pathology framework in which PD-associated cognitive impairment reflects variable contributions from neurotransmitter dysfunction, Lewy-related pathology, Alzheimer-type co-pathology, and distributed network vulnerability rather than nigrostriatal dopamine loss alone [5,6,7,8].

Within this complex pathological background, redox imbalance may provide an important mechanistic link between proteinopathic, inflammatory, mitochondrial, and synaptic disturbances that increase the vulnerability of cognition-relevant neural systems [10,11]. The brain is particularly susceptible to oxidative injury because of its high metabolic demand, abundance of oxidizable lipids, and relatively limited antioxidant reserve [12]. Mitochondria and NADPH oxidase (NOX) enzymes are among the principal endogenous sources of reactive oxygen species (ROS) in the central nervous system [11,13]. Whereas mitochondrial ROS arise partly as byproducts of oxidative metabolism, ROS generation is the primary catalytic function of NOX enzymes [13,14]. Under physiological conditions, NOX-derived oxidants participate in spatially and temporally regulated redox signaling, synaptic function, and innate immune defense [13,14]. However, under excessive or persistent activation, NOX-related redox signaling can amplify neuroinflammation, α-synuclein-associated pathology, mitochondrial dysfunction, synaptic injury, and neuronal damage [10,11,13]. Therefore, NOX1, NOX2, and NOX4 have emerged as potentially modifiable mediators of oxidative injury in PD [10,11]. Earlier NOX-focused reviews highlighted the broader relevance of these enzymes to PD pathogenesis and neurodegenerative redox biology, but did not provide a study-level synthesis of cognition-linked evidence across individual NOX isoforms, brain regions and cell types, and degrees of experimental directness [10,11].

Recent reviews have examined PD-associated cognitive impairment from complementary perspectives, including hippocampal redox mechanisms [15] and a broader multifactorial account of cognitive decline [16], while a separate NOX4-focused review has addressed oxidative stress mechanisms and therapeutic potential in PD more broadly [17]. However, the directness of evidence linking individual NOX isoforms to cognitive outcomes relevant to PD and the extent to which this evidence varies across brain regions, cell types, and experimental paradigms remain insufficiently resolved.

To address this gap, this evidence-focused narrative review critically synthesizes the roles of NADPH oxidases in PD-associated cognitive impairment on the basis of cognition-linked original studies identified through a structured literature search. Within the core synthesis, studies are appraised according to NOX isoform attribution, brain region or biological sample, cellular localization, manipulation or intervention characteristics, behavioral interpretation, and the directness of the NOX–cognition relationship. The synthesis identifies two emerging evidence domains. Hippocampal NOX4 is associated with memory circuit vulnerability and multiple proteinopathy-related changes, whereas NOX2/gp91phox-associated microglial signaling is implicated more prominently in inflammatory amplification, neuronal and synaptic injury, and iron–lipid oxidative damage. By distinguishing between direct genetic evidence, NOX-targeted pharmacological intervention, partial and supportive observations, and complementary pathway-level evidence, this review clarifies the experimental conditions under which NOX-related oxidative stress is implicated in the development of learning and memory deficits in PD-relevant models. It also distinguishes the core experimental evidence from the human clinicopathological and comparative peripheral biomarker context. Moreover, it delineates the additional validation required to establish cross-model generalizability, human relevance, biomarker utility, and therapeutic applicability.

## 2. Literature Search and Evidence Appraisal

### 2.1. Structured Literature Search

To identify studies for the evidence synthesis in this focused narrative review, a structured literature search was conducted across PubMed/MEDLINE, Web of Science Core Collection, Scopus, and Embase. The search combined terms related to PD, cognitive impairment or cognition-related behavioral outcomes, and NADPH oxidases. The initial search conducted on 24 June 2026 yielded 97 records, and an updated search using the same strategy on 11 August 2026 yielded 99 records. To assess search sensitivity, an expanded search incorporating additional PD-model terms and NOX isoform, subunit, and gene names was performed on 6 October 2026. This expanded search yielded 160 records. The expanded search expression was as follows: (Parkinson* OR MPTP OR 6-OHDA OR 6-hydroxydopamine OR oxidopamine OR rotenone OR paraquat OR maneb) AND (cognit* OR memory OR learning OR dementia OR “Morris water maze” OR “Y-maze” OR “novel object recognition”) AND (“NADPH oxidase” OR NOX1 OR NOX2 OR NOX3 OR NOX4 OR NOX5 OR gp91phox OR p47phox OR p22phox OR p67phox OR p40phox OR CYBB OR CYBA OR NCF1 OR NCF2 OR NCF4). Supplementary Table S1 provides the complete database-specific search strategies, platforms, search fields, applied limits, quantities of records retrieved, and search dates for the expanded search, together with a separate summary of database-specific record counts for the initial, updated, and expanded searches. The expanded search and subsequent reference-list screening did not identify any additional eligible original studies.

### 2.2. Eligibility and Study Selection

Original studies were considered eligible for the core evidence base if they examined a human PD or PDD population or a PD-relevant experimental model and evaluated a NOX–cognition relationship within that PD or PD-relevant setting. Each study was required to include a cognitive outcome or cognition-defined diagnostic status together with a NOX-related assessment. NOX-related assessments included isoform or subunit expression, cellular localization, enzymatic activity, NOX-related redox changes, or genetic or pharmacological manipulation targeting NOX or a NOX-associated pathway.

For inclusion in the core evidence base, animal studies were required to include an explicit learning or memory assessment, such as the Morris water maze (MWM), novel-object recognition (NOR), passive avoidance, the Barnes maze, or another cognition-related task. Two additional original studies were retained for separate contextual characterization: a human postmortem study examining NOX4-associated pathology in PD and a canine study examining the association between plasma NOX4 and cognitive–behavioral status outside a PD cohort. The human study did not compare advanced PD cases with and without dementia or report individual cognitive scores, whereas cognition was not assessed in the 1-methyl-4-phenyl-1,2,3,6-tetrahydropyridine (MPTP)-treated comparator group in the canine study. These studies were therefore distinguished from the core evidence base and retained as human clinicopathological and comparative peripheral biomarker context, respectively. Neither established a NOX–cognition association in human PD.

Review articles, editorials, conference abstracts, in vitro-only studies, studies of neurological conditions unrelated to PD, and reports lacking either a cognitive outcome or a NOX-related assessment were excluded from the core evidence base. Review articles were used to inform the background, support reference-list screening, and situate the eligible original studies within the broader literature. Two authors (D.-H.C. and I.-A.C.) independently screened the retrieved records and assessed potentially eligible full-text reports. Screening decisions were compared, and any discrepancies were resolved through discussion and consensus.

The expanded searches yielded 160 records. After removal of 56 duplicate records, 104 unique records remained. Forty-six review articles were excluded from the core evidence base, leaving 58 reports for relevance assessment. Forty-six reports did not meet one or more of the eligibility criteria, and 12 studies—comprising 10 rodent studies with cognitive assessments in PD-relevant models and 2 contextual studies— were retained for study-level synthesis. Supplementary Table S2 lists the excluded reports and principal reasons for exclusion. Supplementary Figure S1 summarizes the literature identification and study-selection process.

### 2.3. Data Extraction

For each study retained for study-level synthesis, we extracted information on the study design, population or experimental model, sample size and, where applicable, animal age and sex, disease induction procedure, and experimental manipulation or intervention. The timing of genetic and pharmacological interventions relative to disease induction and cognitive assessment was recorded because pretreatment, exposure-phase intervention, and delayed post-insult treatment support different mechanistic and therapeutic interpretations.

Cognition-specific assessments and their principal findings were extracted separately from motor, sensory, motivational, and exploratory measures used to evaluate potential behavioral confounding. For animal studies, these additional measures included swimming speed, visible-platform performance, open-field activity, rotational behavior, rotarod performance, and other task-relevant controls when reported.

The NOX-related information included the isoform or subunit examined, assay method, brain region or biological sample, cellular localization, enzymatic activity, and use of genetic or pharmacological manipulation. Redox, inflammatory, neuronal, synaptic, proteinopathy-related, iron-related, and ferroptosis-related outcomes were extracted when they contributed to interpretation of the NOX–cognition relationship.

The study-level appraisal also considered manipulation specificity, sampling context, cellular attribution, intervention timing, reported sample size, and potential behavioral confounding. Whether randomization and blinded outcome assessment were reported was also recorded for the intervention studies, together with relevant behavioral controls. When these methodological features were not reported in the available article or its Supplementary Materials, they were recorded as not reported rather than inferred. Full texts, including figures and figure legends, were examined. Supplementary Materials were also examined when available. Supplementary Table S3 presents the experimental conditions, cognitive outcomes, behavioral confound assessments, NOX-related measurements, intervention characteristics, reporting of randomization and blinded outcome assessment, and study-specific limitations in detail.

### 2.4. Evidence Classification and Narrative Synthesis

The 10 studies constituting the core evidence base were appraised and categorized primarily according to how directly NOX involvement was tested in relation to cognitive outcomes. In cases where an in vivo intervention was directed at NOX, genetic manipulation was distinguished from NOX-targeted pharmacological interventions, whereas complementary mechanistic experiments were considered separately when NOX dependence was demonstrated outside the cognition-tested in vivo paradigm.

The primary study-level categories comprised direct genetic evidence, NOX-targeted pharmacological intervention, partial evidence, and supportive evidence. Within the supportive category, supportive discordant evidence was used when a NOX-related measure did not exhibit a consistent relationship with cognitive status across the comparisons examined. Complementary pathway-level evidence was used as a separate category to capture a distinct form of mechanistic support provided by complementary experiments. This author-defined categorization was developed specifically for this review; it was not derived from an established formal system for grading mechanistic evidence. The categories were descriptive rather than ordinal and were not intended to be an overall assessment of methodological quality, a formal risk-of-bias grade, an assessment of evidence certainty, or a quantitative evidence score. The findings were synthesized narratively, and no quantitative pooling was performed.

Direct genetic evidence was assigned when a specific NOX isoform or NOX complex component was genetically suppressed or deleted in vivo and cognitive outcomes were assessed within the same experimental design. In this category, “direct” refers to direct manipulation of the NOX target in relation to the cognitive phenotype and does not, by itself, establish that accompanying downstream molecular changes mediate the cognitive effect. NOX-targeted pharmacological intervention was assigned when a pharmacological agent that was used explicitly to inhibit NOX was administered in vivo and cognitive outcomes were assessed within the same experimental design. This category reflects the intervention strategy rather than target-specific mediation, and it does not imply isoform, cell-type, or target specificity or establish that the cognitive effect was mediated specifically or exclusively through NOX inhibition. Partial evidence was assigned when cognitive outcomes and NOX-related findings were linked within the same PD-relevant study, but NOX-dependent cognitive causality was not directly tested in vivo, and NOX dependence was not established in complementary mechanistic experiments. This category included upstream vulnerability paradigms and broad intervention studies in which the NOX component was supported by NOX activity measurements, regionally relevant findings, or multiple convergent NOX- and redox-related measures.

Supportive evidence was assigned when the NOX–cognition relationship was based on an isolated NOX-component measurement, including a single-subunit change accompanying a broad intervention with multiple biological effects, without functional validation or additional convergent evidence of NOX involvement. Supportive discordant evidence was assigned when a NOX-related measure did not exhibit a consistent relationship with cognitive status across the comparisons examined. An inverse association alone was not considered discordant.

Complementary pathway-level evidence was assigned when cognition-related pathway components were tested in vivo and NOX dependence was demonstrated in complementary cellular or mechanistic experiments, without direct in vivo manipulation of NOX during cognitive assessment. This category indicates mechanistic support for a NOX-related pathway but does not establish that NOX itself mediated the in vivo behavioral effect.

The human postmortem and canine biomarker studies were retained as contextual studies and evaluated separately from the 10 studies constituting the core evidence base. Study-level information was extracted from these studies to characterize their findings and limitations, but the studies were not assigned the evidence categories used for the 10 core studies.

The narrative synthesis addressed NOX4-related redox changes and cognitive vulnerability, NOX2/gp91phox and related microglial NOX signaling, and NOX activity and subunit-level findings. An integrated appraisal then examined the mechanistic implications and experimental support across these domains.

Additional publications beyond the 12 studies retained for study-level synthesis were used to frame the clinical spectrum and neuropathological basis of PD-associated cognitive impairment and the broader biology of NOX and redox signaling. They were also used to position this review relative to the previous literature and inform the clinical, mechanistic, methodological, and translational interpretations of the core evidence base. These additional publications were used primarily in the Introduction and Discussion and were not included in study-level data extraction or evidence categorization. They were not used to assign or modify evidence categories or establish NOX–cognition causality.

## 3. NOX-Related Mechanisms in PD-Associated Cognitive Impairment

The NOX–cognition findings from the 10 core studies were synthesized across three domains reflecting the NOX isoform or component examined and the level of mechanistic detail provided by the corresponding measurements. These domains comprise NOX4-related findings, NOX2/gp91phox and related microglial NOX signaling, and NOX activity and subunit-level findings. The human postmortem and canine biomarker studies were retained separately as contextual observations and not included as a fourth mechanistic domain. Table 1 summarizes all 12 studies, grouping the 10 core studies across the three mechanistic domains and presenting the 2 contextual studies separately in Panel D.

### 3.1. NOX4-Related Redox Changes and Cognitive Vulnerability

Within the core evidence base, hippocampal NOX4 suppression attenuated learning and memory deficits and proteinopathy-related changes in a PD-relevant 6-OHDA model [18]. Human postmortem and canine biomarker studies provide complementary clinicopathological and peripheral biomarker context, respectively [28,29], but do not establish a NOX4–cognition relationship in human PD.

A direct experimental link between hippocampal NOX4 and the development of learning and memory deficits was demonstrated in a unilateral medial forebrain bundle 6-hydroxydopamine (6-OHDA) model. Nigrostriatal injury induced a dose-dependent impairment in the MWM, together with an increase in hippocampal NOX4, phosphorylated α-synuclein, amyloid-β, and A11-reactive oligomeric species. The regional NOX response was selective. Hippocampal NOX1 and NOX2 remained unchanged, whereas NOX1 levels increased in the substantia nigra, supporting the selective relevance of NOX4 to the hippocampal changes accompanying learning and memory deficits [18]. Functional involvement was examined by delivering an adeno-associated virus (AAV) serotype 2 vector encoding NOX4 short hairpin RNA (shRNA) to the dentate gyrus 3 weeks before 6-OHDA administration. This approach reduced hippocampal NOX4 messenger RNA (mRNA) levels, protein levels, and immunoreactivity and lowered total hippocampal NOX activity. NOX4 knockdown attenuated spatial learning deficits and selected probe-trial abnormalities and improved NOR. Swimming speed was unchanged, and apomorphine-induced rotation and rotarod deficits did not elicit any improvements, reducing the likelihood that the behavioral improvement resulted secondarily from motor recovery. The intervention also attenuated hippocampal phosphorylated α-synuclein, amyloid-β, and A11-reactive oligomeric changes [18]. These results provide direct genetic evidence that NOX4 within the dentate gyrus contributes to the development of learning and memory deficits after nigrostriatal injury. Because knockdown was established before lesion induction, the findings demonstrate protection against the development of learning and memory deficits rather than reversal of an established deficit.

Human postmortem data provide clinicopathological context for NOX4-associated oxidative pathology in PD. Substantia nigra tissue was examined from seven age-matched controls, three neuropathologically defined presymptomatic PD cases without motor symptoms, and five advanced PD cases. Four of the advanced PD cases were recorded as PD with dementia, and one was recorded as PD with concomitant Alzheimer’s disease [28]. Among surviving tyrosine hydroxylase-positive neurons, the proportion of nuclei showing NOX4 immunoreactivity increased from 51% in age-matched controls to 89% in presymptomatic PD and 93% in advanced PD. Nuclear oxidative nucleic acid damage showed a similar pattern, with 8-hydroxyguanosine-positive nuclei increasing from 31% to 68% in presymptomatic and 82% in advanced PD. More intense active caspase-3 immunoreactivity was also observed in the disease groups [28]. These findings reveal greater nuclear NOX4 immunoreactivity together with oxidative nucleic acid damage and apoptotic signaling across presymptomatic PD and advanced PD groups in human nigral dopaminergic neurons. Because NOX4-related pathology was already exacerbated in the presymptomatic PD cases, individual cognitive scores were unavailable, no advanced PD groups without dementia were included, and one advanced case had concomitant AD, the study does not distinguish a cognition-specific NOX4 association from effects related to disease stage or co-pathology. It is therefore interpreted here as human clinicopathological context rather than evidence of a NOX4–cognition relationship in human PD [28].

A comparative canine study provides peripheral biomarker context. In 85 dogs classified as cognitively normal or having mild or severe cognitive impairment using a modified canine cognitive dysfunction rating scale, plasma NOX4 concentrations were lower in the cognitively impaired groups and inversely associated with cognitive–behavioral severity [29]. A machine learning model using plasma NOX4 and retinol-binding protein 4 distinguished cognitively impaired from cognitively normal dogs [29]. The cognitively impaired groups were older, and age-related effects may have contributed to the observed NOX4–cognition association. The canine cohort was not a PD cohort, and cognition was not assessed in the separate MPTP-treated mouse comparator [29]. These findings therefore provide comparative peripheral biomarker context rather than PD-specific evidence of a NOX4–cognition relationship, and the relationship between circulating and central NOX4 biology remains to be established.

Taken together, the core experimental evidence supports the notion that hippocampal NOX4 contributes to the development of learning and memory deficits after nigrostriatal injury [18]. The human postmortem and canine studies broaden the clinicopathological and peripheral biomarker context of NOX4, respectively [28,29], but do not establish a NOX4–cognition association in human PD.

### 3.2. NOX2/gp91phox and Related Microglial NOX Signaling

Microglial NOX2/gp91phox signaling is implicated in learning and memory deficits accompanied by microglial activation, pro-inflammatory responses, and neuronal and synaptic injury in PD-relevant models [19,20,21,22,23,24]. Apocynin treatment and gp91phox loss-of-function can attenuate learning and memory deficits [19,24]. Noradrenergic dysfunction and microglial inflammatory pathways have also been linked to NOX-associated cognitive vulnerability [20,21,22,23]. In selected models, NOX2-associated pathology was accompanied by iron dysregulation and lipid oxidative injury, including ferroptosis-related changes in the rotenone model [20,24].

#### 3.2.1. NOX-Targeted Pharmacological Intervention and Learning and Memory Outcomes

Apocynin was used to examine the contribution of NOX-related processes to learning and memory deficits in a paraquat-plus-maneb (P+M) mouse model [19]. Repeated P+M exposure in 3-month-old male C57BL/6J mice induced progressive deficits in spatial learning and memory, with abnormalities in MWM acquisition and probe trial performance becoming evident after 6 weeks. These behavioral changes occurred together with hippocampal and cortical neuronal injury, reduced postsynaptic density protein 95 (PSD-95) levels, and increased hippocampal α-synuclein expression and Ser129 phosphorylation. Administration of apocynin in drinking water began 2 days before the first pesticide exposure and continued throughout the 6-week exposure period. The treatment improved acquisition performance and increased time spent in the target quadrant without altering swimming speed, although the reduction in first platform-crossing latency did not reach statistical significance [19].

The behavioral effects of apocynin were accompanied by reductions in hippocampal gp91phox and p47phox expression. Oxidative changes were also attenuated, with reduced superoxide-related signals assessed by water-soluble tetrazolium salt-1 reduction, lower dihydroethidium oxidation, and decreased malondialdehyde and lipid hydroperoxide levels. Complementary BV2 experiments also showed attenuation of p47phox membrane translocation. In vivo, apocynin reduced microglial activation assessed via ionized calcium-binding adaptor molecule 1 (Iba-1) and cluster of differentiation 11b (CD11b) immunostaining along with pro-inflammatory gene expression. It also attenuated signal transducer and activator of transcription 1 and nuclear factor kappa B signaling, preserved neuronal and synaptic markers, and ameliorated α-synuclein-related pathology [19]. These behavioral and molecular changes are consistent with involvement of NOX-related oxidative and inflammatory processes in the learning and memory deficits observed. Because apocynin is nonselective, however, the findings do not establish that the behavioral benefit was mediated specifically by microglial NOX2 or exclusively by NOX inhibition [19]. Its administration before pesticide exposure further indicates protection during the development of learning and memory deficits rather than reversal of an established impairment.

#### 3.2.2. Noradrenergic Dysfunction and NOX2-Associated Cognitive Vulnerability

Two studies using the noradrenergic neurotoxin DSP-4 examined noradrenergic dysfunction as an upstream condition that may increase susceptibility to NOX2-associated inflammatory and oxidative injury. In a 4-week P+M exposure paradigm, 3-month-old male mice were administered DSP-4 1 week before the first P+M administration. After 4 weeks of P+M exposure, neither P+M alone nor DSP-4 alone induced significant deficits in MWM acquisition or probe-trial performance, whereas combined DSP-4 and P+M exposure impaired acquisition and multiple probe-trial measures without altering swimming speed [20]. The combined exposure also triggered reductions in hippocampal neuronal nuclei (NeuN) and PSD-95 and increased total and Ser129-phosphorylated α-synuclein levels. These changes were accompanied by iron accumulation, altered iron-handling proteins, reduced glutathione peroxidase 4 (GPX4) and glutathione levels, and increased lipid peroxidation [20].

Within the combined DSP-4 and P+M condition, learning and memory impairment co-occurred with elevated hippocampal p47phox and gp91phox expression, prominent colocalization of gp91phox with Iba-1-positive microglia, microglial activation assessed via Iba-1 immunostaining, and a pro-inflammatory transcript profile [20]. These findings link noradrenergic disruption to hippocampal NOX2-associated microglial changes and inflammatory and iron–lipid oxidative pathology accompanying learning and memory impairment. Direct NOX activity and NOX-directed rescue were not assessed, so they do not establish that NOX2 mediates the behavioral effect [20].

A complementary DSP-4/lipopolysaccharide (LPS) model was used to examine progressive NOX2-associated inflammatory and oxidative pathology following early noradrenergic disruption. DSP-4 was administered 1 week before systemic LPS exposure, and gp91phox-related pathology was evaluated over 3–10 months. Combined DSP-4 and LPS treatment produced a progressive increase in nigral gp91phox immunoreactivity, with gp91phox localized to Iba-1-positive microglia and accompanied by increasing neuronal 3-nitrotyrosine and 4-hydroxynonenal signals [21]. At 7.5 months after treatment, when the mice were 42 weeks old, hidden platform acquisition, visible platform performance, and swimming speed remained preserved. However, the LPS and DSP-4-plus-LPS groups failed to show target quadrant or former platform location preferences during the MWM probe trial, indicating impaired spatial memory retention [21]. Therefore, the study provides partial evidence that delayed spatial memory impairment is accompanied by NOX2-associated inflammatory and oxidative pathology. Because probe-trial impairment was also present in the LPS-alone group, the behavioral findings do not demonstrate further worsening specifically attributable to DSP-4.

Together, these studies suggest that noradrenergic dysfunction may increase susceptibility to NOX2-associated inflammatory and oxidative pathology. The P+M study links this susceptibility to hippocampal injury and the emergence of learning and memory deficits, whereas the LPS study characterizes progressive microglial gp91phox-related pathology and neuronal oxidative injury in a model with delayed spatial memory impairment [20,21].

#### 3.2.3. Mac1–NOX1/NOX2–NLRP3 Inflammatory Signaling

Taurine treatment was associated with improved learning and memory performance and reduced hippocampal macrophage-1 antigen (Mac1)/Src–extracellular signal-regulated kinase (ERK)/NOX2-associated signaling [22]. In this 6-week P+M paradigm, taurine, administered before each pesticide exposure, improved MWM acquisition and target-quadrant retention without altering swimming speed [22]. Improved MWM performance was accompanied by reduced Iba-1-positive microglial activation, lower gp91phox and p47phox expression and p47phox phosphorylation, reduced CD11b expression and Src–ERK phosphorylation, decreased ROS-related signals, partial restoration of superoxide dismutase activity, and preservation of NeuN and PSD-95 [22]. Because taurine has broad biological effects and the study did not directly manipulate Mac1 or NOX2, these findings support coordinated involvement of NOX2-associated inflammatory and redox signaling but do not establish pathway-specific mediation of the behavioral effects.

In another P+M study, the relationship between Mac1 and NLR family pyrin domain containing 3 (NLRP3) inflammasome signaling was examined in vivo, with complementary experiments assessing NOX1 and NOX2 dependence in cultured microglia [23]. Global Mac1 deletion attenuated P+M-induced MWM acquisition deficits and improved platform-crossing numbers and target quadrant time. These behavioral improvements were accompanied by reduced hippocampal microglial activation, NLRP3 inflammasome-related signaling, and α-synuclein-related pathology, together with attenuated neuronal and synaptic injury in the hippocampus and cortex [23]. Exposure-phase treatment with glybenclamide also reduced NLRP3-related molecular changes and improved acquisition [23].

In parallel, the NOX component was examined primarily in BV2 microglial cells and selected experiments in primary microglia. Mac1 blockade, apocynin, and either NOX1 or NOX2 small interfering RNA (siRNA) reduced P+M-induced NLRP3 inflammasome activation, whereas pharmacological NOX activation with phorbol 12-myristate 13-acetate counteracted the inhibitory effect of Mac1 blockade [23]. These complementary experiments support a model in which Mac1 is upstream of microglial NOX1/NOX2-dependent inflammasome signaling. However, because Mac1 deletion was global and NOX1/NOX2 dependence was established in cultured microglia rather than in the cognition-tested animals, the NOX1/NOX2 dependence of the in vivo learning and memory effects was not directly tested [23].

#### 3.2.4. gp91phox Loss-of-Function, Learning and Memory Outcomes, and Iron–Lipid Oxidative Injury

The contribution of gp91phox to rotenone-induced learning and memory deficits was examined using a loss-of-function approach. Eight-week-old male mice were administered intracerebroventricular AAV carrying CD68-driven gp91phox shRNA; this was followed 4 weeks later by daily rotenone exposure for 21 days. The AAV reporter signal overlapped with Iba-1-positive cells in the hippocampus and cortex, supporting reporter expression in microglia, although exclusive cell-type specificity was not established. Rotenone increased gp91phox expression and p47phox phosphorylation in the hippocampus and cortex, reduced NADPH levels, and increased the NADP^+^/NADPH ratio [24]. Together, these changes are consistent with enhanced NOX2-associated signaling in cognition-relevant brain regions.

CD68-driven gp91phox knockdown attenuated MWM acquisition deficits and improved selected probe trial measures without altering swimming speed. A separate global gp91phox-deficient cohort provided additional genetic support, showing a similar improvement in learning and selected memory outcomes [24]. Together, these complementary loss-of-function findings support the notion that gp91phox played a contributory role in the development of learning and memory deficits in the rotenone model.

Gp91phox knockdown also reduced Iba-1-associated microglial activation and pro-inflammatory transcript abundance and attenuated NeuN loss and PSD-95 reduction in the hippocampus and cortex. In parallel, it reduced tissue iron accumulation and attenuated disruption of iron metabolism proteins and iron-dependent lipid peroxidation while restoring GPX4 mRNA levels and attenuating the upregulation of other ferroptosis-related transcripts [24]. An iron chelation experiment further reduced rotenone-induced lipid peroxidation abnormalities, indicating that the oxidative injury has an iron-dependent component, although cognition was not assessed in that experiment [24]. Thus, improvement in learning and memory after gp91phox knockdown was accompanied by reductions in microglial inflammation, neuronal and synaptic injury, iron dysregulation, and ferroptosis-related changes. However, these concomitant changes were not tested as necessary mediators of the behavioral effects.

Liproxstatin-1 was used to examine whether ferroptosis-related injury contributed to the rotenone-induced learning and memory deficits. Treatment preserved NeuN and PSD-95 and improved MWM acquisition and selected probe trial outcomes [24]. This result supports the notion that ferroptosis-related injury contributes to learning and memory impairment in the rotenone model. However, gp91phox loss-of-function and ferroptosis inhibition were examined in parallel rather than in a sequential causal design, so the study did not establish ferroptosis as a required downstream mediator of the behavioral effects associated with loss of function of gp91phox. With gp91phox knockdown established before rotenone exposure, the study primarily evaluated protection against the development of learning and memory deficits rather than reversal of an established impairment.

Across the six studies, microglial NOX2/gp91phox signaling is consistently associated with learning and memory deficits accompanied by microglial activation, pro-inflammatory signaling, and neuronal and synaptic injury [19,20,21,22,23,24]. NOX-targeted pharmacological interventions and gp91phox loss-of-function mutation attenuated these behavioral deficits, while noradrenergic disruption and Mac1–NOX1/NOX2–NLRP3 signaling revealed upstream conditions and inflammatory pathways that may enhance NOX-associated cognitive vulnerability [19,20,21,22,23,24]. In selected models, iron dysregulation and lipid oxidative injury were also observed, with the rotenone study further implicating ferroptosis-related processes [20,24]. Together, these findings support the notion that NOX2/gp91phox-associated microglial inflammatory redox signaling plays a contributory role in the development of learning and memory deficits in PD-relevant models, while the downstream relationship between gp91phox and ferroptosis remains to be defined more directly.

### 3.3. NOX Activity and Subunit-Level Findings

Alterations in NOX activity and individual NOX subunits have also been examined in relation to learning and memory in PD-relevant models. These studies assessed striatal NOX activity, striatal p47phox protein, and regional p22phox mRNA expression in experiments involving chrysin, vitamin D_3_, and treadmill exercise, respectively [25,26,27].

In 20-month-old female mice with unilateral intrastriatal 6-OHDA lesions, chrysin treatment, initiated 7 days after lesioning and continued for 28 days, improved passive-avoidance and Barnes-maze retention [25]. The behavioral improvement was accompanied by reduced striatal NOX activity, together with attenuation of oxidative and inflammatory abnormalities and partial restoration of striatal dopamine and its metabolites [25]. The assay measured diphenyleneiodonium-sensitive oxidase activity in striatal homogenates without identifying the responsible NOX isoform or cellular source. Chrysin has multiple biological effects, and treatment was accompanied by improvements in apomorphine-induced rotational asymmetry and striatal dopamine metabolism, suggesting that broader attenuation of lesion-related abnormalities may also have contributed to the observed memory improvement [25].

Vitamin D_3_ treatment was initiated 2 weeks after lesioning and improved NOR performance on treatment day 16 [26]. This behavioral improvement occurred in parallel with reduced striatal p47phox protein levels and changes in several inflammatory, neurotrophic, and dopaminergic markers [26]. Because p47phox was the only NOX-related component assessed and vitamin D_3_ has broad biological effects, the study did not establish whether reduced p47phox reflected altered activity of a specific NOX complex or directly accounted for the recognition memory improvement. These parallel behavioral and subunit-level changes therefore provide supportive evidence without establishing NOX-dependent mediation [26].

In an exercise-based MPTP model, habit learning was assessed alongside regional NOX-related gene expression changes. In male Wistar rats aged 10 weeks at the start of the experiment, treadmill exercise initiated before intranasal MPTP administration and continued afterward attenuated the prolonged escape latency observed in sedentary MPTP rats during a cued water-maze task assessing habit learning [27]. NOX-related assessment was limited to p22phox mRNA within a transcriptomic analysis of the frontal cortex, hippocampus, and striatum, with the principal alteration detected in the frontal cortex. Frontal cortical p22phox mRNA levels were lower in sedentary MPTP rats, and they were also reduced by treadmill exercise in saline-treated animals. However, the two MPTP groups differed in habit learning performance without a significant between-group difference in p22phox mRNA expression [27]. This pattern indicates that p22phox transcript abundance did not consistently track habit learning performance across groups. Without corresponding protein or enzyme activity measurements, these findings do not establish whether the transcript changes reflected altered NOX function [27].

Across these studies, functional oxidase activity, subunit protein abundance, and subunit transcript abundance provided distinct levels of mechanistic information. Chrysin- and vitamin D_3_-associated memory improvements coincided with reductions in oxidase activity and p47phox protein levels, respectively, whereas p22phox mRNA did not consistently track habit learning performance in the exercise study [25,26,27]. Although the administration of chrysin and vitamin D_3_ was initiated after lesioning, neither study documented a learning or memory deficit before treatment initiation; therefore, these experiments do not establish reversal of a pre-existing deficit. Because the catalytic isoform responsible and cellular source were not established, these findings extend the range of NOX-related observations associated with learning and memory outcomes without identifying an additional isoform-specific mechanism.

### 3.4. Integrated Evidence Appraisal

Across the 10 studies constituting the core evidence base, the NOX–cognition relationship differed in how directly NOX involvement was tested in relation to cognitive outcomes. Dentate-gyrus-targeted NOX4 knockdown and gp91phox loss of function provided direct genetic evidence [18,24], whereas apocynin was categorized as a NOX-targeted pharmacological intervention because it was administered in vivo with cognitive assessment but lacked isoform, cell-type, and target specificity [19]. The Mac1–NOX1/NOX2–NLRP3 study was categorized as complementary pathway-level evidence because cognition-related Mac1 and NLRP3 signaling was examined in vivo, whereas NOX1/NOX2 dependence was demonstrated in complementary microglial experiments rather than in the cognition-tested animals [23]. The remaining core studies contributed partial, supportive, or supportive discordant evidence through upstream vulnerability paradigms, broad interventions, functional but isoform-unresolved oxidase activity, or individual NOX subunit measurements [20,21,22,25,26,27]. Rather than being assigned the evidence categories used for the 10 core studies, the human postmortem and canine biomarker studies were evaluated separately as contextual studies [28,29].

When considered across these categories, the most direct experimental support is concentrated in two evidence domains involving hippocampal NOX4 and NOX2/gp91phox-associated microglial signaling [18,24]. NOX4 knockdown attenuated the development of learning and memory deficits together with hippocampal proteinopathy-related changes, whereas gp91phox loss of function attenuated learning and memory deficits together with reductions in microglial inflammation, neuronal and synaptic injury, and iron–lipid oxidative damage [18,24]. These concomitant molecular changes were not tested as necessary mediators of the behavioral effects.

**Figure 1:**
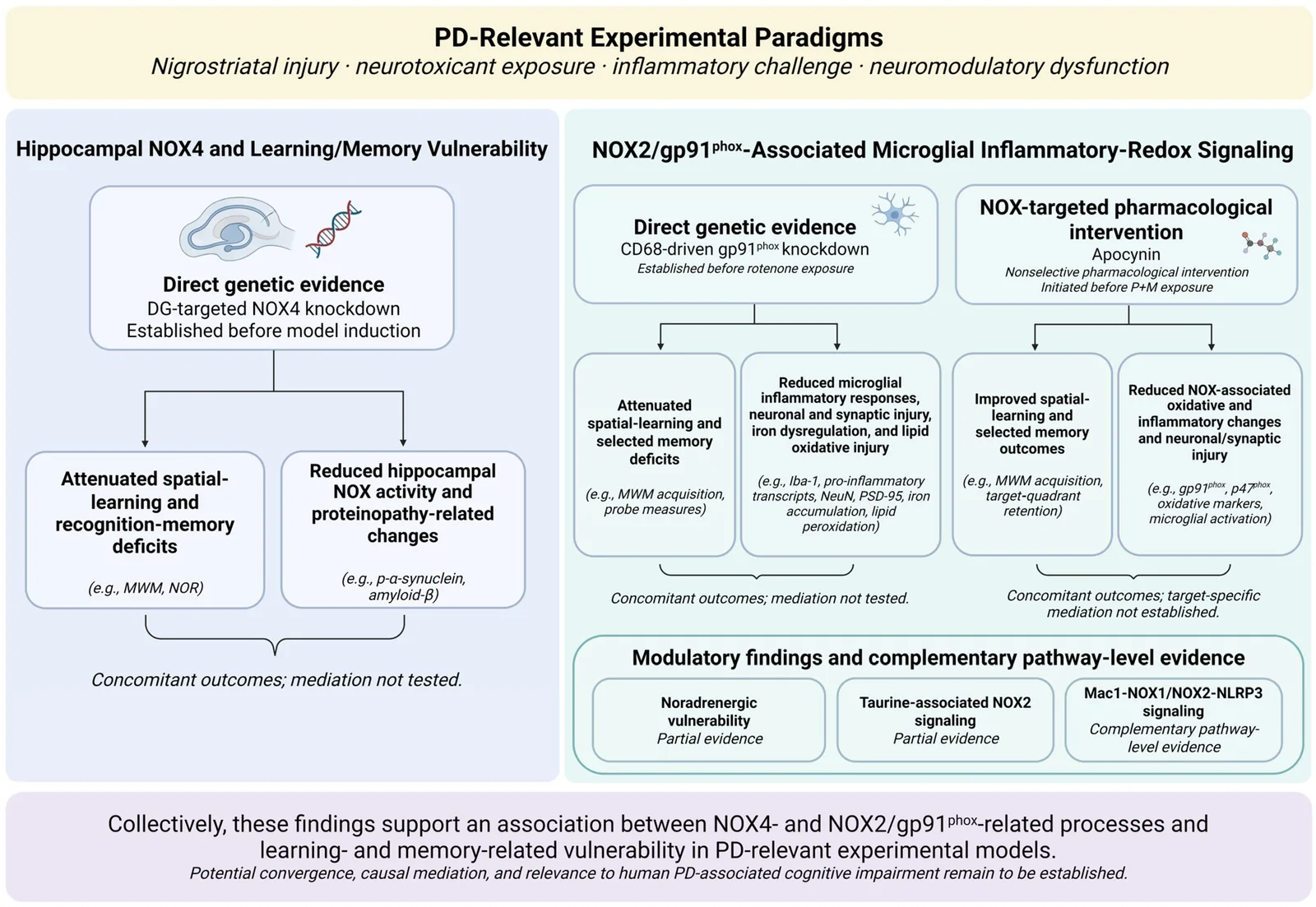
Conceptual synthesis of the two principal NOX-related evidence domains associated with learning and memory deficits in PD-relevant experimental models. The figure summarizes the hippocampal NOX4 study [18] and the NOX2/gp91phox-associated experimental evidence, including the apocynin intervention study [19], the noradrenergic-vulnerability studies [20,21], the taurine-associated NOX2 signaling study [22], the Mac1-NOX1/NOX2-NLRP3 pathway study [23], and the gp91phox loss-of-function study [24]. Additional core studies addressing isoform-unresolved NOX activity or individual NOX subunits [25,26,27] are summarized in Table 1 and Table 2 and are not depicted here. The figure was created using BioRender. Choi, I. (2026). Available online: https://BioRender.com/md2nl58, accessed on 15 October 2026.

Overall, the core evidence supports the notion that NOX-related oxidative and inflammatory signaling play a contributory role in the development of learning and memory deficits in the PD-relevant experimental models examined, while the extent of mechanistic support differs across NOX isoforms, biological compartments, and experimental designs. The human postmortem and canine biomarker studies provide complementary clinicopathological and peripheral biomarker context, respectively, but do not establish a NOX–cognition association in human PD [28,29]. Table 2 summarizes the principal mechanistic themes, supporting findings, and key validation requirements. Figure 1 summarizes the two principal NOX-related evidence domains identified in the core experimental literature.

## 4. Discussion

### 4.1. Potential Mechanistic Convergence of NOX4- and NOX2/gp91phox-Associated Processes in Cognitive Vulnerability

The integrated evidence suggests that NOX-related oxidative stress contributes to cognitive vulnerability in PD-relevant experimental models through region- and cell-dependent mechanisms rather than through a single uniform pathway [18,24]. The relevance of these mechanisms to PD-associated cognitive impairment can be understood through their potential interactions with α-synuclein accumulation, Alzheimer-type co-pathology, mitochondrial dysfunction, neuroinflammation, neurotransmitter system degeneration, and synaptic and network disruption [6,7,8,15,16]. This interpretation is consistent with the spatially and temporally regulated nature of redox signaling and the distinct activation properties and cellular distributions of individual NOX isoforms [10,11,13,14]. The consequences of NOX-derived oxidant production are therefore likely to depend on its cellular source and regulation rather than on increased oxidant production alone.

Dentate-gyrus-targeted NOX4 loss of function attenuated learning and memory deficits without improving motor abnormalities and reduced hippocampal phosphorylated α-synuclein, amyloid-β, and A11-reactive oligomeric species [18]. Together with the reduction in hippocampal NADPH oxidase activity, these findings support the involvement of hippocampal NOX4 in the development of the behavioral phenotype [18]. However, the associated redox and proteinopathy-related changes were not tested as necessary mediators of the behavioral effect. Redox dysregulation can affect protein folding and clearance, mitochondrial function, and synaptic integrity, while proteinopathic and mitochondrial disturbances can further increase oxidant burden [10,11,15,17]. Evidence from non-PD tauopathy models, in which NOX4 suppression improved autophagic–lysosomal function and reduced pathological tau accumulation, further links NOX4 to the regulation of protein clearance [13,17,30]. Conversely, experimental studies showing that α-synuclein oligomers induce oxidative stress and impair mitochondrial function provide a basis for considering protein accumulation as both a consequence and a source of cellular oxidative injury [15,17,31]. Hippocampal NOX4 may therefore contribute to reciprocal interactions between oxidative stress, impaired protein homeostasis, and mitochondrial dysfunction that increase the vulnerability of memory circuitry [10,15,17,18].

Microglial NOX2/gp91phox is implicated in inflammatory signaling and oxidative injury. CD68-driven gp91phox knockdown attenuated learning and memory deficits and was accompanied by reductions in microglial activation and neuronal and synaptic injury. Reporter colocalization with Iba-1-positive cells supported microglial involvement, although exclusive cell-type specificity was not established [24]. Broader experimental studies show that extracellular aggregated α-synuclein can activate microglial NADPH oxidase and ROS production, providing a route through which protein pathology may recruit glial oxidative responses [10,11,32]. Loss of noradrenergic restraint may increase susceptibility to NOX2-associated inflammatory and oxidative responses [20,21], whereas Mac1-related signaling provides a potential upstream route for microglial NOX1/NOX2-dependent inflammasome activation in cultured microglia [23]. These findings suggest that altered neuromodulatory regulation and receptor-mediated inflammatory signaling can influence the susceptibility of neural tissue to NOX-associated injury. Improved learning and memory performance after apocynin or taurine treatment, accompanied by reductions in NOX-associated inflammatory changes, is consistent with this interpretation [19,22]. The rotenone findings further link gp91phox to iron dysregulation, lipid oxidative injury, and ferroptosis-related changes [24], although these concomitant changes were not established as necessary mediators of the behavioral effects. Thus, microglial NOX2/gp91phox may participate in an amplification process connecting inflammatory activation to neuronal, synaptic, and lipid oxidative injury.

Hippocampal NOX4-related redox dysregulation accompanied by proteinopathy-related abnormalities and NOX2/gp91phox-associated microglial inflammatory signaling can therefore be viewed as complementary, nonexclusive mechanisms contributing to the dysfunction of cognition-relevant neural systems. α-Synuclein-related abnormalities are observed across several studies involving NOX4- or NOX2-associated signaling [18,19,20,23]. The broader relationships between protein accumulation, glial oxidant production, and impaired protein clearance provide a biological basis for considering convergence between these processes [10,15,17]. Such convergence may occur through overlapping effects on neuronal protein homeostasis and synaptic function, without requiring the two isoforms to have identical cellular distributions or downstream actions. NOX-related oxidative stress is therefore interpreted as an interacting component of a multi-pathological process, with its cognitive consequences shaped by brain region, cellular source, and coexisting pathological processes [6,7,8,10,15,16,18,24].

### 4.2. Evidence Scope and Generalizability

Within the core evidence base, the rodent studies used toxin-, lesion-, or inflammatory-challenge paradigms that reproduce selected pathological features of PD rather than its full biological heterogeneity [18,19,20,21,22,23,24,25,26,27]. This model concentration is most evident in the NOX2/gp91phox studies, where four of six studies used related P+M paradigms [19,20,22,23]. Although these studies addressed different questions about NOX2-associated inflammation and oxidative injury, their shared use of P+M supports convergence within that model rather than validation across pathobiologically distinct PD models. Independent replication of the hippocampal NOX4 and CD68-driven gp91phox loss-of-function findings could be performed to assess the consistency of the reported learning and memory effects [18,24]. Cross-model validation should be used to determine whether NOX4 or gp91phox suppression also attenuates learning and memory deficits in progressive models driven primarily by α-synuclein pathology.

Most rodent studies used male animals, with model induction generally beginning in young adulthood, while aged animals and females were underrepresented [18,19,20,21,22,23,24,25,26,27]. Comparisons of young-adult and aged animals of both sexes would allow determination of whether the behavioral effects of NOX4 or gp91phox suppression persist across age and sex. Consistent with the animal study eligibility criteria, cognitive assessment emphasized learning and memory, with the MWM used in seven of the 10 rodent studies and in all six studies contributing to the NOX2/gp91phox domain [18,19,20,21,22,23,24]. Additional tasks included NOR, passive avoidance, the Barnes maze, and a cued water maze [18,25,26,27]. Accordingly, the current experimental evidence primarily provides support for effects on learning and memory rather than the broader spectrum of cognitive impairment in PD. Cognitive batteries should therefore incorporate tasks assessing attention, executive function, cognitive flexibility, working memory, and visuospatial processing to extend coverage of cognitive domains relevant to PD-MCI and PDD. Task-relevant behavioral controls should accompany these assessments. In particular, unchanged swimming speed alone does not exclude all noncognitive influences on water-maze performance.

The mechanistic interpretation of the evidence also depends on the type of NOX-related measurement used. NOX-directed genetic loss-of-function tests the manipulated target’s contribution to the behavioral phenotype, whereas changes in isoform or subunit expression, phosphorylation, enzyme activity, and downstream oxidative injury represent related but non-equivalent levels of biological information. The authors of other studies used whole-tissue measurements, individual NOX components, or isoform-unresolved oxidase activity, providing less-precise attribution of the catalytic isoform, cellular source, or both [19,20,21,22,25,26,27]. In the exercise study, p22phox mRNA did not consistently track habit learning performance across the comparisons examined, and corresponding protein expression and enzyme activity were not assessed. The transcript findings therefore did not establish whether NOX function was altered [27]. In functional validation, researchers should therefore pair regional and cellular targeting of a defined NOX isoform with measurements of NOX activity or NOX-derived oxidant production in cognition-relevant circuits. These measurements would test whether target suppression alters the redox response associated with the behavioral phenotype.

For Mac1-associated microglial signaling, in vivo comparisons of NOX1 and NOX2 suppression within the same cognitive paradigm, combined with selective manipulation of Mac1 and NLRP3 signaling, would clarify the isoform-specific contributions and the relationships between these components [23]. Sequential loss-of-function, rescue, or epistasis experiments could distinguish mediating processes from parallel pathological responses. For example, a candidate downstream process could be reactivated after NOX suppression to determine whether the behavioral improvement is attenuated or reversed, with controls for the effects of reactivation alone. Such experiments could assess whether the behavioral effects of NOX modulation require NOX4-associated hippocampal proteinopathy, NLRP3 signaling, or gp91phox-associated iron-dependent lipid oxidative injury [18,23,24].

### 4.3. Translational Relevance and Therapeutic Implications

Hippocampal NOX4 knockdown and CD68-driven gp91phox knockdown attenuated learning and memory deficits, while apocynin provided additional, nonselective pharmacological support for the involvement of NOX-related processes [18,19,24]. These findings provide an experimental rationale for further therapeutic investigation of NOX-related mechanisms, with target selection informed by the implicated isoform, brain region, and cellular context.

Human observations provide clinicopathological context for NOX-related pathology in PD but do not establish a NOX–cognition relationship in human PD. Nigral NOX4-associated oxidative pathology was observed both in advanced PD cases with dementia or concomitant Alzheimer’s disease and in neuropathologically defined presymptomatic PD cases without motor symptoms [28]. However, the study in question did not include an advanced PD group without dementia or relate NOX4 measurements to individual cognitive scores. The inclusion of a case with concomitant AD further limits separation of a cognition-specific NOX4 association from effects exerted by disease stage or co-pathology. Beyond the 12 studies retained for study-level synthesis, human postmortem studies have also reported increased gp91phox/NOX2 expression in nigral microglia [33] and increased NOX2 activation in both nigral dopaminergic neurons and microglia, as assessed via p47phox–NOX2 proximity ligation [34]. These observations support the relevance of NOX-related pathology to human PD, but they were not linked to characterized cognitive status. Prospective cohorts spanning PD with preserved cognition, PD-MCI, and PDD should combine standardized cognitive phenotyping with NOX4- and NOX2/gp91phox-related measurements. Postmortem analyses should extend beyond the substantia nigra to hippocampal, limbic, and cortical regions and relate regional and cellular NOX expression or activity to documented premortem cognitive status.

The canine study provides comparative peripheral biomarker context rather than PD-specific evidence. Plasma NOX4 concentrations were lower in cognitively impaired dogs and inversely associated with cognitive–behavioral severity. A machine learning model combining NOX4 and retinol-binding protein 4 distinguished cognitively impaired dogs from cognitively normal ones [29]. The cognitively impaired groups were older, and age-related effects may have contributed to the observed association [29]. Biomarker development should be conducted to distinguish between a peripheral correlate of cognitive status and an indicator of central NOX4 activity, because the relationship between circulating NOX4 and brain NOX4 expression or function remains undefined. Whether circulating NOX4 is relevant to cognitive impairment in human PD therefore remains to be established. Validation studies on human PD should separately evaluate associations with current cognitive status, prediction of subsequent decline, and suitability for treatment monitoring. Use as an indicator of central target engagement would additionally require evidence that treatment-related biomarker changes reflect modulation of the intended brain NOX target.

Taurine, chrysin, and vitamin D_3_ improved learning and memory outcomes together with changes in NOX-related and broader inflammatory, oxidative, dopaminergic, metabolic, or neurotrophic processes [22,25,26]. These findings indicate that pathological processes associated with NOX signaling are modifiable, while the extent to which NOX modulation contributed to the behavioral improvements induced by these broader interventions remains unresolved. Intervention timing is a major limitation of the current therapeutic evidence. The principal NOX4 and gp91phox genetic interventions were established before model induction and therefore primarily support protection during the development of learning and memory deficits rather than reversal of an already-established impairment [18,24]. Apocynin treatment was likewise initiated before P+M exposure [19], while chrysin and vitamin D_3_ treatment began after lesion induction [25,26]. However, learning or memory impairment was not demonstrated before either treatment was initiated, so these post-lesion paradigms also do not establish reversal of established cognitive impairment. Therapeutic validations should therefore include inducible or pharmacological NOX-directed interventions initiated after cognitive impairment has been confirmed. Repeated cognitive assessment, with pretreatment baselines and concurrent control groups, would distinguish attenuation of further decline from improvement in established deficits.

Beyond the cognition-linked evidence base, the brain-penetrant NOX2 inhibitor CPP11G reduced NOX2 activation and lipid peroxidation in nigrostriatal dopaminergic neurons during rotenone exposure, with brain exposure confirmed after oral administration [34]. Although cognition was not assessed, this study provides complementary evidence that pharmacological NOX2 inhibition is feasible in the central nervous system. Therefore, it supports pharmacological targetability but does not provide evidence for treatment of established cognitive impairment. Preclinical pharmacological development should establish dose–exposure–response relationships linking administered doses and brain exposure to inhibition of the intended NOX target and cognitive outcomes. Assessment of isoform selectivity and effects in the relevant cell populations would help determine whether behavioral improvement is associated with the intended target modulation.

Because NOX-derived ROS also participate in physiological redox signaling, synaptic regulation, and immune function [11,13,14], the therapeutic goal is likely to be selective attenuation of pathological NOX activity rather than broad suppression of ROS generation. Isoform selectivity alone cannot distinguish physiological from pathological NOX activity, so the degree and duration of inhibition also require consideration. Taken together, the current evidence identifies hippocampal NOX4 and NOX2/gp91phox-associated microglial signaling as priorities for further target validation. Their therapeutic relevance will require interventions initiated after cognitive impairment has been confirmed and direct evaluation in cognitively characterized PD populations.

### 4.4. Strengths and Limitations of the Present Review

A principal strength of this review is its integration of a structured four-database search incorporating a broad range of PD-model- and NOX-related terms with a critical synthesis of cognition-linked NOX findings in PD. Study-level appraisal of the 12 studies retained for synthesis, comprising 10 core rodent studies and 2 contextual studies, considered cognitive outcomes, NOX isoform or component, brain region or biological sample, cellular localization, and manipulation or intervention characteristics and timing. For the 10 core studies, the author-defined evidence categorization primarily reflects how directly NOX involvement was tested in relation to cognitive outcomes. In cases where an in vivo intervention was directed at NOX, genetic manipulation was distinguished from NOX-targeted pharmacological intervention, while complementary mechanistic evidence was considered separately when NOX dependence was demonstrated outside the cognition-tested in vivo paradigm. This categorization was developed for this review and was descriptive rather than ordinal; it should be regarded as a structured interpretive approach rather than a formal system for grading evidence quality or certainty. The appraisal also considered selected methodological features but did not include formal risk-of-bias or evidence-certainty assessment. Randomization of the relevant main intervention or comparison cohort was explicitly reported in seven of the 10 core studies. Blinded behavioral assessment was reported for at least some outcomes in three studies, whereas blinded histological or immunohistochemical assessment was reported in two. Blinding with respect to molecular assays was generally not reported. These findings reflect the completeness of methodological reporting and should not be interpreted as evidence that unreported procedures were not performed.

The relatively small and heterogeneous evidence base limited cross-study comparability. The predominance of toxin- and lesion-based models, the concentration of behavioral evidence on learning and memory, and the absence of cognition-linked human NOX validation further limit generalization to the broader clinical spectrum of PD-associated cognitive impairment. These limitations emphasize the need for replication across distinct disease models, broader cognitive phenotyping, and cognitively characterized human PD cohorts.

## 5. Conclusions

The evidence reviewed herein supports the notion that NOX-related oxidative stress contributes to the development of learning and memory deficits in PD-relevant experimental models. The most direct mechanistic support concerns hippocampal NOX4 and gp91phox-associated microglial signaling. Dentate-gyrus-targeted NOX4 loss of function attenuated learning and memory deficits and was accompanied by reductions in hippocampal proteinopathy-related changes, whereas CD68-driven gp91phox knockdown attenuated learning and memory deficits and was accompanied by reductions in microglial inflammation, neuronal and synaptic injuries, iron dysregulation, and lipid oxidative damage [18,24]. These accompanying molecular changes were not established as necessary mediators of the behavioral effects. A NOX-targeted pharmacological intervention with apocynin provides additional support for the involvement of NOX-related processes, although its lack of target and isoform specificity limits attribution of the behavioral effect specifically to NOX inhibition [19]. Pathway and noradrenergic vulnerability studies further link NOX-associated inflammatory and oxidative processes to learning and memory deficits [20,21,22,23]. Activity- and subunit-level findings provide additional but less direct information on NOX-related changes associated with behavioral outcomes [25,26,27].

Importantly, the principal NOX4 and gp91phox genetic interventions were established before model induction and therefore primarily support protection during the development of learning and memory deficits rather than reversal of established cognitive impairment. Human nigral NOX4-associated oxidative pathology and the inverse association between canine plasma NOX4 and cognitive–behavioral severity provide human clinicopathological and comparative peripheral biomarker context, respectively, but do not establish a NOX–cognition relationship in human PD [28,29]. Cross-model reproducibility, NOX-directed interventions initiated after cognitive impairment, has been demonstrated, and validation of NOX-related measures in cognitively characterized human PD cohorts is needed in order to determine the applicability of these findings to biomarker development and selective therapeutic targeting.

## Figures and Tables

**Table 1:** Study characteristics and evidence categories for NOX-related findings relevant to Parkinson’s disease-associated cognitive impairment.

**A. NOX4-Related Redox Changes and Cognitive Vulnerability**					
**Study**	**Population/model**	**Cognitive assessment**	**NOX assessment**	**Manipulation/intervention**	**Key findings and evidence categories**
Choi et al., 2019 [18]	Male C57BL/6 mice, 12 wk; unilateral MFB 6-OHDA	MWM; NOR	Hippocampal NOX4; total NOX activity	DG-targeted AAV2–shNOX4	Development of learning and memory deficits was attenuated without motor recovery. Direct genetic evidence
**B. NOX2/gp91phox and Related Microglial NOX Signaling**					
**Study**	**Population/model**	**Cognitive assessment**	**NOX assessment**	**Manipulation/intervention**	**Key findings and evidence categories**
Hou et al., 2019 [19]	Male C57BL/6J mice, 3 mo; P+M	MWM	Hippocampal gp91phox/p47phox	Apocynin	Apocynin pretreatment attenuated learning and memory deficits and was accompanied by reductions in NOX-related oxidative and inflammatory changes. NOX-targeted pharmacological intervention
Hou et al., 2019 [20]	Male C57BL/6J mice, 3 mo; P+M	MWM	Hippocampal gp91phox and p47phox	DSP-4 pretreatment	DSP-4 pretreatment unmasked learning and memory deficits during P+M exposure, along with hippocampal NOX2-associated pathology. Partial evidence
Song et al., 2019 [21]	Male C57BL/6J mice, 9 wk; LPS	MWM	Nigral microglial gp91phox	DSP-4 pretreatment	Delayed spatial memory impairment accompanied gp91phox-associated inflammatory and oxidative pathology. Partial evidence
Wang et al., 2021 [22]	Male C57BL/6J mice, 12 wk; P+M	MWM	Hippocampal gp91phox, p47phox, and p-p47phox	Taurine	Taurine-associated improvement in learning and memory performance coincided with reduced hippocampal NOX2-associated signaling. Partial evidence
Hou et al., 2023 [23]	Male C57BL/6 and Mac1-/- mice, 8 wk; P+M	MWM	NOX1/NOX2 dependence in cultured microglia	Mac1 deletion in vivo; apocynin and NOX1/NOX2 siRNA in vitro	Mac1 deletion improved learning and memory performance in vivo, while complementary microglial experiments demonstrated the NOX1/NOX2 dependence of NLRP3 activation. Complementary pathway-level evidence
Tian et al., 2024 [24]	Male C57BL/6 mice, 8 wk; rotenone	MWM	Hippocampal and cortical gp91phox and p-p47phox	CD68-driven AAV–shgp91phox; global gp91phox knockout	Gp91phox loss-of-function attenuated the development of learning and memory deficits. Direct genetic evidence
**C. NOX Activity and Subunit** **-Level Findings**					
**Study**	**Population/model**	**Cognitive assessment**	**NOX assessment**	**Manipulation/intervention**	**Key findings and evidence categories**
Del Fabbro et al., 2019 [25]	Female C57BL/6J mice, 20 mo; unilateral striatal 6-OHDA	Passive avoidance; Barnes maze	Striatal NOX activity	Chrysin	Memory improvement accompanied reduced subtype-unresolved striatal NOX activity. Partial evidence
Bayo-Olugbami et al., 2022 [26]	Male C57BL/6 mice, 13 wk; unilateral striatal 6-OHDA	NOR	Striatal p47phox protein	VD_3_	Recognition memory improvement occurred with reduced striatal p47phox. Supportive evidence
Viana et al., 2017 [27]	Male Wistar rats, 10 wk; intranasal MPTP	Cued water maze	Frontal cortical p22phox mRNA	Treadmill exercise	p22phox mRNA did not consistently track habit learning performance across the comparisons examined. Supportive discordant evidence
**D. Contextual Human Clinicopathological and Comparative Biomarker Findings**					
**Study**	**Population/model**	**Cognitive assessment**	**NOX assessment**	**Manipulation/intervention**	**Key findings and evidence categories**
Zawada et al., 2015 [28]	Human postmortem SN; age-matched controls, presymptomatic PD, and advanced PD; advanced PD included 4 PD/dementia and 1 PD/AD	Clinical dementia diagnosis	Nuclear NOX4 in nigral dopaminergic neurons	—	Nigral neuronal nuclear NOX4 and oxidative nucleic acid damage were higher in presymptomatic and advanced PD. Human clinicopathological context
Kim et al., 2024 [29]	Normal/MCI/SCI dogs, mean age—7/12/15 y, 41M/44F; separate MPTP-treated mouse comparator without cognitive assessment	Modified CCDR	Plasma NOX4 protein	—	Lower plasma NOX4 was associated with greater CCDR severity. Comparative peripheral biomarker context

The 10 studies constituting the core evidence base are grouped into three mechanistic domains, and the two contextual studies are presented separately in Panel D. For experimental studies, ages refer to the first experimental manipulation unless otherwise specified. Evidence categories are descriptive rather than ordinal, and they are defined in Section 2.4. Complementary pathway-level evidence denotes in vivo testing of cognition-related pathway components combined with complementary mechanistic demonstration of NOX dependence without direct in vivo NOX manipulation during cognitive assessment. The two contextual studies were not assigned the evidence categories used for the 10 core studies. In the human postmortem study, dementia-associated diagnostic status was available, but individual cognitive scores were not reported. In the canine study, cognition was assessed in the canine cohort but not in the separate MPTP-treated mouse comparator. Supportive discordant evidence denotes findings in which the NOX-related measure did not exhibit a consistent relationship with cognitive status across the comparisons examined. Cognitive assessments in the 10 core rodent studies primarily reflect learning and memory domains rather than the broader spectrum of cognitive impairment in PD. Detailed experimental conditions and study-specific limitations are provided in Supplementary Table S3—no experimental manipulation or intervention. Abbreviations: 6-OHDA, 6-hydroxydopamine; AD, Alzheimer’s disease; AAV, adeno-associated virus; CCDR, canine cognitive dysfunction rating; DG, dentate gyrus; DSP-4, N-(2-chloroethyl)-N-ethyl-2-bromobenzylamine hydrochloride; F, female; LPS, lipopolysaccharide; M, male; Mac1, macrophage-1 antigen; MCI, mild cognitive impairment; MFB, medial forebrain bundle; MPTP, 1-methyl-4-phenyl-1,2,3,6-tetrahydropyridine; MWM, Morris water maze; NLRP3, NLR family pyrin domain containing 3; NOR, novel object recognition; NOX, NADPH oxidase; P+M, paraquat plus maneb; PD, Parkinson’s disease; p-p47phox, phosphorylated p47phox; SCI, severe cognitive impairment; SN, substantia nigra; VD_3_, vitamin D_3_; wk, weeks; mo, months; y, years.

**Table 2:** Mechanistic synthesis of NOX-related findings and key validation requirements in Parkinson’s disease-associated cognitive impairment.

Mechanistic Theme	Principal Findings	Additional Findings	Key Validation Requirements
Hippocampal NOX4 and cognitive vulnerability	DG-targeted NOX4 knockdown attenuated spatial learning and recognition memory deficits without improving lesion-related motor abnormalities in a 6-OHDA model [18].	Human nigral NOX4-associated oxidative pathology in presymptomatic and advanced PD and the inverse association between canine plasma NOX4 and cognitive–behavioral severity provide clinicopathological and peripheral biomarker context, respectively, without establishing a NOX4–cognition relationship in human PD [28,29].	Direct NOX4-derived oxidant production, cross-model replication, intervention after learning and memory deficits are established, and quantitative cognition–NOX4 relationships in human PD remain to be established.
Microglial NOX2/gp91phox and cognitive injury	CD68-driven gp91phox knockdown improved selected learning and memory outcomes and reduced inflammatory, neuronal, and synaptic injury; reporter colocalization supported microglial involvement, and global gp91phox deletion provided additional genetic support [24].	Apocynin attenuated learning and memory deficits together with gp91phox/p47phox-associated oxidative, inflammatory, and synaptic abnormalities [19].	Functional NOX2 activity or oxidant production in cognition-relevant regions, cross-model validation including aged animals of both sexes and progressive α-synuclein models, intervention after learning and memory deficits have been established, and cognition-linked human validation remain priorities.
Loss of noradrenergic restraint and NOX2-associated vulnerability	DSP-4 pretreatment was associated with learning and memory impairment and hippocampal injury during a shorter P+M exposure that did not produce clear behavioral deficits with either challenge alone [20].	In a progressive DSP-4/LPS model, noradrenergic dysfunction accelerated gp91phox-associated oxidative pathology and neurodegeneration, although spatial memory retention was not more impaired than with LPS alone [21].	Direct in vivo testing is needed to determine whether NOX2 mediates the cognitive vulnerability associated with LC/NE dysfunction and distinguish inflammatory from direct noradrenergic effects on cognition.
Mac1–NOX1/NOX2–NLRP3 inflammatory signaling	Global Mac1 deletion improved MWM performance and reduced NLRP3 activation in vivo, while NOX1 or NOX2 silencing suppressed inflammasome activation in cultured microglia [23].	Taurine-associated improvement in MWM performance occurred with reduced hippocampal CD11b/Src–ERK and NOX2-associated signaling [22].	In vivo isoform-specific NOX1/NOX2 manipulation during cognitive testing and sequential validation of Mac1–NOX–NLRP3 relationships are needed.
NOX2-associated iron and lipid oxidative injury	Gp91phox knockdown reduced iron accumulation and lipid oxidative injury, while a parallel liproxstatin-1 intervention improved neuronal, synaptic, and selected learning and memory outcomes [24].	DSP-4/P+M exposure induced iron dysregulation, reductions in GPX4/GSH, increased lipid peroxidation, and NOX2-associated subunit changes in the hippocampus [20].	Sequential causal testing is needed to determine whether ferroptosis-related injury mediates the behavioral effects associated with gp91phox loss of function rather than occurring as a parallel pathological process.
NOX activity and subunit-level findings	Chrysin improved memory together with reduced subtype-unresolved striatal NADPH oxidase activity in aged female 6-OHDA mice [25].	Vitamin D_3_ improved NOR performance together with reduced striatal p47phox, whereas frontal cortical p22phox transcription did not induce a parallel exercise-associated habit learning improvement [26,27].	Isoform and cellular attribution remain unresolved, and activity, protein, and transcript measures require interpretation according to the extent to which they reflect functional NOX signaling.

The principal findings summarize the key observations for each mechanistic theme, while additional findings broaden or qualify their interpretation. These columns indicate the role of findings within each theme rather than their evidence category. Key validation requirements indicate unresolved mechanistic or translational questions. Detailed study characteristics and evidence categories for the 10 core studies, together with the two contextual studies, are provided in Table 1. Study-specific limitations and reporting notes are provided in Supplementary Table S3. Abbreviations: 6-OHDA, 6-hydroxydopamine; DG, dentate gyrus; DSP-4, N-(2-chloroethyl)-N-ethyl-2-bromobenzylamine hydrochloride; ERK, extracellular-signal-regulated kinase; GPX4, glutathione peroxidase 4; GSH, glutathione; LC, locus coeruleus; LPS, lipopolysaccharide; Mac1, macrophage-1 antigen; NE, norepinephrine; NLRP3, NLR family pyrin domain containing 3; NOR, novel-object recognition; NOX, NADPH oxidase; P+M, paraquat plus maneb; PD, Parkinson’s disease; VD_3_, vitamin D_3_.

## Data Availability

No new research data were generated in this study. All information supporting this review is available in the cited publications and the Supplementary Materials.

## Supplementary Materials

The following supporting information can be downloaded at https://www.mdpi.com/article/10.3390/antiox15101367/s1: Figure S1: Flow diagram of research identification and study selection. Table S1: Database search strategies and records retrieved. Table S2: Reports excluded from study-level synthesis and primary reasons for exclusion [35,36,37,38,39,40,41,42,43,44,45,46,47,48,49,50,51,52,53,54,55,56,57,58,59,60,61,62,63,64,65,66,67,68,69,70,71,72,73,74,75,76,77,78,79]. Table S3: Detailed study design, NOX-related evidence, and methodological appraisal of the studies retained for study-level synthesis.

## Abbreviations

The following abbreviations are used in this manuscript:

6-OHDA	6-hydroxydopamine
AAV	adeno-associated virus
AD	Alzheimer’s disease
CCDR	canine cognitive dysfunction rating
CD11b	cluster of differentiation 11b
CD68	cluster of differentiation 68
DG	dentate gyrus
DSP-4	N-(2-chloroethyl)-N-ethyl-2-bromobenzylamine hydrochloride
ERK	extracellular signal-regulated kinase
GPX4	glutathione peroxidase 4
GSH	glutathione
Iba-1	ionized calcium-binding adaptor molecule 1
LC	locus coeruleus
LPS	lipopolysaccharide
Mac1	macrophage-1 antigen
MCI	mild cognitive impairment
MFB	medial forebrain bundle
MPTP	1-methyl-4-phenyl-1,2,3,6-tetrahydropyridine
mRNA	messenger RNA
MWM	Morris water maze
NADP^+^	oxidized nicotinamide adenine dinucleotide phosphate
NADPH	reduced nicotinamide adenine dinucleotide phosphate
NE	norepinephrine
NeuN	neuronal nuclei
NLRP3	NLR family pyrin domain containing 3
NOR	novel object recognition
NOX	NADPH oxidase
P+M	paraquat plus maneb
PD	Parkinson’s disease
PD-MCI	Parkinson’s disease mild cognitive impairment
PDD	Parkinson’s disease dementia
PSD-95	postsynaptic density protein 95
ROS	reactive oxygen species
SCI	severe cognitive impairment
shRNA	short hairpin RNA
siRNA	small interfering RNA
SN	substantia nigra
