# Supplementary material for: NADPH Oxidase-Related Oxidative Stress in Parkinson’s Disease-Associated Cognitive Impairment: An Evidence-Focused Review
Source: Antioxidants (Basel). 2026 Oct 21;15(10):1367. doi: 10.3390/antiox15101367 (PMC13701224; doi:10.3390/antiox15101367)
Supplement: Supplementary file 1 [file antioxidants-15-01367-s001.zip › Revised Figure S1-update.pdf]

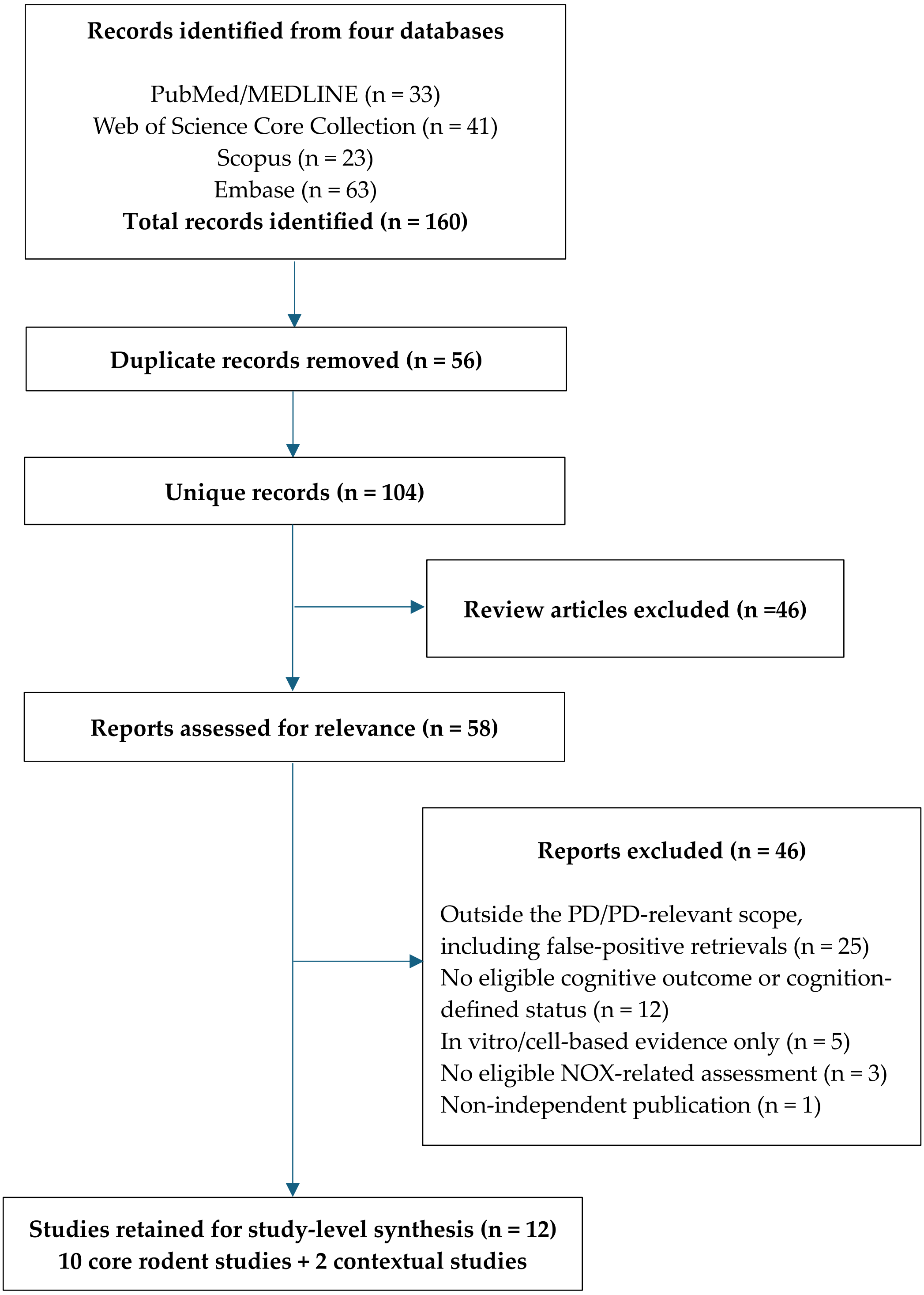

Supplementary Figure S1. Flow diagram of research identification and study selection. The expanded searches of PubMed/MEDLINE, Web of Science Core Collection, Scopus, and Embase identified 160 records. After removal of 56 duplicates, 104 unique records remained. Forty-six review articles were excluded before report-level relevance assessment. Of the 58 reports assessed for relevance, 46 did not meet the eligibility criteria, leaving 12 studies for study-level synthesis, comprising 10 core rodent studies and two contextual studies. Detailed report-level exclusion reasons are provided in Supplementary Table S2.
