# Supplementary material for: NADPH Oxidase-Related Oxidative Stress in Parkinson’s Disease-Associated Cognitive Impairment: An Evidence-Focused Review
Source: Antioxidants (Basel). 2026 Oct 21;15(10):1367. doi: 10.3390/antiox15101367 (PMC13701224; doi:10.3390/antiox15101367)
Supplement: Supplementary file 1 [file antioxidants-15-01367-s001.zip › Revised Table S3.pdf]

Supplementary Table S3. Detailed study design, NOX-related evidence, and methodological appraisal of the studies retained for study-level synthesis

Panel A. Study design, cognitive assessment, and behavioral controls

Study	Population/model and groups	Disease induction or exposure	Manipulation/intervention and timing	Cognitive assessment and principal result	Behavioral confound assessment
**Choi et al., 2019 [18]**	Male C57BL/6 mice, 12 wk. Dose-response 6-OHDA cohort and four-group AAV cohort (sham/6-OHDA × scrambled/NOX4-shRNA); n=12/group for behavior and n=6/group for molecular analyses in the AAV cohort	Unilateral medial forebrain bundle (MFB) 6-OHDA lesion; 3 μg used in the AAV cohort; outcomes assessed 4 wk after lesioning.	Bilateral dentate gyrus (DG) AAV2-shNOX4 or scrambled AAV2 administered 3 wk before 6-OHDA to establish stable knockdown	Morris water maze (MWM) and novel object recognition (NOR). NOX4 knockdown improved acquisition/selected probe measures and NOR discrimination.	Swimming speed was unchanged. NOX4 knockdown did not improve apomorphine-induced rotation or rotarod deficits. Visible-platform performance was assessed in the initial model-characterization cohort.
**Hou et al., 2019 [19]**	Male C57BL/6J mice, 3 mo. Time-course cohorts: control and P+M, n=45/group; MWM n=8–15/group/time point. Apocynin experiment included control, P+M, apocynin+P+M, and apocynin-alone groups	Paraquat 10 mg/kg plus maneb 30 mg/kg i.p., twice weekly for 6 wk.	Apocynin 1 mg/mL in drinking water (estimated ~150 mg/kg/day), initiated 2 d before the first P+M exposure and continued for 6 wk	MWM at 2, 4, and 6 wk. Deficits emerged at 6 wk. Apocynin improved acquisition and target-quadrant time; first platform-crossing latency was not significantly restored.	Swimming speed was unchanged; no additional task-relevant behavioral control was reported for the apocynin intervention experiment.
**Hou et al., 2019 [20]**	Male C57BL/6J mice, 3 mo; control, P+M, DSP-4, and DSP-4+P+M groups. Initial n=10-15/group; MWM n=8-12/group; molecular analyses n=4-6/group	Paraquat 10 mg/kg plus maneb 30 mg/kg i.p., twice weekly for 4 wk, initiated 1 wk after DSP-4 pretreatment.	DSP-4 50 mg/kg i.p. once, administered 1 wk before P+M exposure to establish noradrenergic vulnerability; no NOX-directed intervention	MWM. P+M or DSP-4 alone showed no clear deficit at 4 wk; the combined DSP-4 and P+M group showed impaired acquisition and probe performance.	Swimming speed was unchanged across groups; no additional task-relevant behavioral control was reported.
**Song et al., 2019 [21]**	Male C57BL/6J mice; DSP-4 at 9 wk and LPS at 10 wk; MWM n=6/group at 42 wk	Single DSP-4 50 mg/kg i.p., followed 1 wk later by LPS 15×10^6 EU/kg i.p.; vehicle, DSP-4, LPS, and combined DSP-4 and LPS groups.	DSP-4 pretreatment to model noradrenergic dysfunction; no NOX-directed intervention	Visible- and hidden-platform MWM. Spatial acquisition remained intact, whereas the LPS and combined DSP-4 and LPS groups lacked target-quadrant and former-platform-location preference. The combined treatment was not demonstrably worse than LPS alone.	Visible-platform performance and swimming speed were preserved; rotarod, wire-hang, and gait were also assessed longitudinally.
**Wang et al., 2021 [22]**	Male C57BL/6J mice, 12 wk; control, P+M, and taurine+P+M groups. Initial n=12-15/group; MWM n=10/group; immunohistochemistry n=6/group	Paraquat 10 mg/kg plus maneb 30 mg/kg i.p., twice weekly for 6 wk	Taurine 150 mg/kg i.p. administered before each P+M exposure for 6 wk	MWM. Taurine improved acquisition and target-quadrant time; platform-crossing number was not improved.	Swimming speed was unchanged; no additional task-relevant behavioral control was reported.
**Hou et al., 2023 [23]**	Male C57BL/6 and global Mac1-/- mice, 8 wk; WT/KO vehicle and P+M groups. Mac1 MWM n=13-16/group; glybenclamide MWM n=15-16/group; molecular analyses typically n=3-6/group	Paraquat 10 mg/kg plus maneb 30 mg/kg i.p., twice weekly for 6 wk	Global Mac1 deletion; glybenclamide 1 mg/kg i.p. 30 min before each P+M exposure; complementary microglial experiments with Mac1 blockade, apocynin, PMA, and NOX1/NOX2 siRNA	MWM. Mac1 deletion improved acquisition, platform crossings, and target-quadrant time; first-crossing latency was unchanged. Glybenclamide improved acquisition; probe outcomes were not reported.	Swimming speed was unchanged in both the Mac1 genetic and glybenclamide experiments; no additional task-relevant behavioral control was reported.

Panel A. Study design, cognitive assessment, and behavioral controls (continued)

Study	Population/model and groups	Disease induction or exposure	Manipulation/intervention and timing	Cognitive assessment and principal result	Behavioral confound assessment
**Tian et al., 2024 [24]**	Main AAV cohort: male C57BL/6 mice, 8 wk, n=16/group (MWM n=15–16). Supporting global gp91phox-deficient cohort: n=11–13/group; liproxstatin-1 cohort: n=15/group	Rotenone 1.5 mg/kg/day i.p. for 21 d, starting 4 wk after AAV delivery	Intraventricular AAV-CD68-shgp91phox or control AAV administration 4 wk before rotenone; supporting global gp91phox deletion; complementary iron-chelator (DFO) and liproxstatin-1 interventions during rotenone exposure	MWM. AAV knockdown improved acquisition, first-crossing latency, and platform-crossing number, but not target-quadrant time. Global deletion and liproxstatin-1 provided additional support for selected outcomes.	Swimming speed was unchanged in the AAV, global gp91phox-deficient, and liproxstatin-1 cohorts; no additional task-relevant behavioral control was reported.
**Del Fabbro et al., 2019 [25]**	Female C57BL/6J mice, 20 mo, 30-40 g; four groups, n=10/group. Passive avoidance/open field/rotation n=5/group; Barnes maze n=10/group; biochemical analyses n=4-5/group	Unilateral right intrastriatal 6-OHDA, 5 μg in 2 μL	Chrysin 10 mg/kg/day intragastrically, initiated 7 d after lesioning and continued for 28 d	Passive avoidance (15-min and 24-h retention) and Barnes maze (4-d acquisition; day-5/day-12 probes). Chrysin improved passive-avoidance memory and day-12 Barnes-maze retention; acquisition was not impaired.	Open-field distance traveled was unchanged, whereas apomorphine-induced rotation improved with chrysin.
**Bayo-Olugbami et al., 2022 [26]**	Male C57BL/6 mice, 13 wk, mean 27.5 g. Of 54 lesioned mice, 40 met lesion criteria; plus 10 sham mice. Five groups, n=10/group; behavioral subset n=5–6/group	Unilateral right intrastriatal 6-OHDA, 3 μg in 2 μL, after desipramine/pargyline; lesion screening by cylinder and apomorphine rotation	VD_3 30 mg/kg/day s.c. from 2 wk after lesioning for 21 d; L-DOPA 10 mg/kg/day plus benserazide 7.5 mg/kg, alone or combined with VD_3	NOR with a 60-min interval on treatment days 8 and 16. VD_3 improved the memory index at day 16 but not day 8.	Cylinder, horizontal-bar, open-field, rearing, and freezing behaviors were assessed; VD_3 improved several motor and exploratory measures.
**Viana et al., 2017 [27]**	Male Wistar rats, 10 wk; sedentary saline, treadmill saline, sedentary MPTP, and treadmill MPTP groups, n=11/group	Intranasal MPTP, 1 mg/nostril (2 mg total), after 30 d of treadmill training; behavior assessed 6-10 d after MPTP and tissue collected at 12 d	Mild treadmill exercise, 60 min/day, 5 d/wk for 6 wk; initiated 30 d before MPTP and continued after exposure	Cued water maze assessing habit learning. MPTP prolonged escape latency, whereas exercise improved performance.	Open-field spontaneous locomotion and swimming speed were unchanged; splash-test performance was also assessed and improved with exercise.
**Zawada et al., 2015 [28]**	Human postmortem substantia nigra: age-matched controls n=7 (67-87 y; 6M/1F), prePD n=3 (69-86 y; 3M), advanced PD n=5 (66-85 y; 3M/2F; 4 PD/dementia and 1 PD/AD)	Clinicopathological groups; no experimental disease induction.	None	Cognitive status was ascertained from clinical diagnoses. Individual cognitive tests, scores, and dementia diagnostic criteria were not reported.	Not applicable.
**Kim et al., 2024 [29]**	Client-owned dogs n=85: normal n=37 (7±3.5 y; 16M/21F), MCI n=25 (12±3.0 y; 14M/11F), SCI n=23 (15±2.7 y; 11M/12F). Separate biomarker-development cohort: male C57BL/6J mice, 7-8 wk, n=20 (MPTP n=10)	Canine groups defined by a modified canine cognitive dysfunction rating (CCDR) scale. MPTP 30 mg/kg/day i.p. for 30 d was used in the comparative mouse workflow.	None in the canine cohort	Modified 10-item CCDR. Plasma NOX4 was lower with greater cognitive-behavioral severity; cognition was not assessed in the MPTP cohort.	Age differed markedly across cognitive groups; CCDR was owner-reported, and some dogs had neurological conditions that could influence behavioral ratings.

Panel B. NOX-related measurements, mechanistic readouts, evidence interpretation, and limitations

Study	Brain region/sample and cellular localization	NOX assessment	Associated mechanistic readouts	NOX-cognition interpretation / evidence category	Main limitations and reporting notes
**Choi et al., 2019 [18]**	Hippocampal DG; substantia nigra examined separately. NOX4 localization was consistent with neuronal-rich DG regions	NOX4 mRNA, protein, and immunoreactivity; total hippocampal NADPH oxidase activity; hippocampal NOX1/NOX2 unchanged; nigral NOX1 increased	Phosphorylated α-synuclein, Aβ, and A11-reactive oligomeric species	DG-targeted NOX4 loss-of-function attenuated learning and memory deficits; Direct genetic evidence	NOX4 knockdown was established before 6-OHDA lesioning and therefore does not establish reversal of established cognitive impairment; direct NOX4-derived oxidant production was not measured. Randomization of the AAV cohort was not reported. Blinding was reported for apomorphine rotation, NOR, and histological quantification, but not for MWM or molecular assays.
**Hou et al., 2019 [19]**	Hippocampus and cortex in vivo; BV2 microglia for p47phox translocation	Hippocampal gp91phox and p47phox expression; superoxide-related WST-1 reduction and DHE oxidation; BV2 p47phox membrane translocation	MDA and lipid hydroperoxides; Iba-1/CD11b, pro-inflammatory transcripts, STAT1/NF-κB, NeuN, PSD-95, TH, and total/p-α-synuclein	Apocynin improved learning and memory performance and was accompanied by reductions in NOX-related oxidative and inflammatory injury; NOX-targeted pharmacological intervention	Apocynin is nonselective, limiting isoform- and cell-specific attribution; treatment began before P+M exposure and therefore does not establish reversal of established cognitive impairment. Randomization was reported for the separate time-course cohort but not for the apocynin intervention cohort; blinded outcome assessment was not reported. A 2025 corrigendum replaced representative PSD-95 and CD11b images; the authors reported that the study conclusions were unchanged.
**Hou et al., 2019 [20]**	Hippocampus; qualitative gp91phox–Iba-1 colocalization in the combined DSP-4 and P+M group	Hippocampal gp91phox and p47phox protein expression	NeuN, PSD-95, total/p-α-synuclein, iron and iron-handling proteins, GPX4, GSH, MDA, Iba-1, and inflammatory transcripts	DSP-4 pretreatment unmasked learning and memory deficits during P+M exposure, accompanied by hippocampal NOX2-associated pathology; Partial evidence	No NOX-directed manipulation or activity assay was performed; NOX2 mediation of the learning and memory phenotype was not directly tested. Although gp91phox and p47phox were elevated in the combined DSP-4 and P+M group, their additional increase relative to P+M alone was not statistically significant. Randomization was reported; blinded outcome assessment was not reported.
**Song et al., 2019 [21]**	Substantia nigra: gp91phox–Iba-1 colocalization; hippocampus and motor cortex: microglial activation, oxidative injury, and neuronal loss	Gp91phox immunoreactivity	Iba-1/CD11b, neuronal 3-NT and 4-HNE, and regional neuronal loss	Delayed spatial-memory deficits accompanied gp91phox-associated inflammatory and oxidative pathology; Partial evidence	Gp91phox localization was demonstrated most clearly in substantia nigra rather than in cognition-relevant hippocampal or cortical regions, and gp91phox-related pathology was not assessed at the late MWM time point. No NOX-directed manipulation was performed, and the combined DSP-4 and LPS group was not demonstrably more impaired than LPS alone. Randomization was not reported. Behavioral data collection was blinded, whereas blinding of histological or molecular assessment was not reported.
**Wang et al., 2021 [22]**	Whole hippocampal lysates; Iba-1 immunohistochemistry assessed separately	Gp91phox, p47phox, and phosphorylated p47phox protein	CD11b, Src–ERK phosphorylation, Iba-1, ROS/SOD-related measures, NeuN, active caspase-3, and PSD-95	Taurine-associated improvement in learning and memory performance coincided with reduced hippocampal NOX2-associated signaling; Partial evidence	Taurine has broad biological effects, and Mac1/NOX2 signaling was not directly manipulated; NOX-related measurements were obtained from whole hippocampal tissue rather than a defined cellular source. Randomization was reported. Blinded assessment was reported for NeuN, Iba-1, and PSD-95 immunohistochemistry, but not for behavioral or other molecular assessments.
**Hou et al., 2023 [23]**	In vivo hippocampus and cortex; NOX mechanistic perturbations conducted in BV2 and selected primary microglial experiments	Apocynin, PMA, and NOX1/NOX2 siRNA in cultured microglia	Mac1, Iba-1, NLRP3, active caspase-1, mature IL-1β, NeuN, PSD-95, TH, total/p-α-synuclein, MAPK, and PAK1	Mac1 deletion improved learning and memory performance in vivo, while NOX1/NOX2 dependence was demonstrated in cultured microglia; Complementary pathway-level evidence	Mac1 deletion was global rather than microglia-specific, and NOX1/NOX2 dependence was established in cultured microglia rather than in the cognition-tested animals. Randomization was reported for the principal WT/Mac1−/− cohort but not for the glybenclamide cohort; blinded outcome assessment was not reported.

Panel B. NOX-related measurements, mechanistic readouts, evidence interpretation, and limitations (continued)

Study	Brain region/sample and cellular localization	NOX assessment	Associated mechanistic readouts	NOX-cognition interpretation / evidence category	Main limitations and reporting notes
**Tian et al., 2024 [24]**	Hippocampus and cortex; CD68-driven AAV reporter overlapped with Iba-1-positive cells, supporting microglial involvement	Gp91phox protein, p47phox phosphorylation, NADPH and $NADP^+$/NADPH ratio; AAV-CD68-shgp91phox and supporting global gp91phox deletion	Iba-1 and inflammatory transcripts; NeuN/PSD-95; tissue iron and iron-handling markers; MDA and GSSG/GSH; ferroptosis-related markers; DFO and liproxstatin-1 interventions	CD68-driven gp91phox loss-of-function attenuated learning and selected memory deficits; Direct genetic evidence, with additional support from global gp91phox deletion	Gp91phox knockdown was established before rotenone exposure and therefore does not establish reversal of established cognitive impairment. Reporter colocalization with Iba-1-positive cells supported microglial involvement but did not establish exclusive cell-type specificity. Gp91phox loss-of-function and ferroptosis inhibition were examined in parallel; therefore, ferroptosis-related changes were not established as necessary mediators of the behavioral effects. Randomization was reported for the main AAV cohort but not for the supporting global gp91phox-deficient or liproxstatin-1 cohorts; blinded outcome assessment was not reported.
**Del Fabbro et al., 2019 [25]**	Striatal homogenate	Striatal NADPH oxidase activity	Nitrite/nitrate, GSH, HNE, reactive species, Na^+/K^+-ATPase activity, dopamine and metabolites, IL-1β, and TNF-α	Memory improvement accompanied reduced subtype-unresolved striatal NADPH oxidase activity; Partial evidence	Chrysin has broad biological effects, and the NADPH oxidase activity assay did not identify the responsible NOX isoform or cellular source. Although treatment began after 6-OHDA lesioning, learning or memory impairment was not demonstrated before treatment initiation. Randomization was reported; blinded outcome assessment was not reported.
**Bayo-Olugbami et al., 2022 [26]**	Whole striatal lysate	p47phox protein	CD11b, IL-1β, BDNF, TH, DAT, MAO-B, COMT, DDC, and BAX	Recognition-memory improvement occurred with reduced striatal p47phox; Supportive evidence	NOX-related assessment was limited to p47phox protein, and the same objects were reused for NOR testing on days 8 and 16. Although treatment began after lesion verification, learning or memory impairment was not demonstrated before treatment initiation. Randomization was reported after lesion screening; blinded outcome assessment was not reported.
**Viana et al., 2017 [27]**	Frontal cortex, hippocampus, and striatum; the principal p22phox transcriptional change was detected in frontal cortex	p22phox mRNA	RAGE/TLR-related and cytokine transcripts, DJ-1, and motivational behavior	p22phox mRNA did not consistently track habit-learning performance across the comparisons examined; Supportive discordant evidence	NOX assessment was limited to p22phox mRNA, and the cued water-maze task primarily assessed habit learning. Exercise began before MPTP exposure and continued thereafter, so the study does not establish reversal of established cognitive impairment. Randomization and blinded behavioral video analysis were reported; blinding of molecular assessment was not reported.
**Zawada et al., 2015 [28]**	Substantia nigra matrix and nigrosome 1; nuclear localization in TH-positive dopaminergic neurons	Nuclear NOX4 immunofluorescence	nuclear 8-hydroxyguanosine and active caspase-3	Nigral neuronal nuclear NOX4 and oxidative nucleic-acid damage were increased in presymptomatic and advanced PD; Human clinicopathological context	No advanced PD group without dementia was included; individual cognitive scores and dementia diagnostic criteria were not reported; NOX4 activity was not measured.
**Kim et al., 2024 [29]**	Peripheral canine plasma; separate MPTP mouse serum biomarker workflow	Plasma NOX4 protein by immunoblotting and ELISA	RBP4 and CXCL10; machine-learning classification of cognitive-behavioral groups	Lower plasma NOX4 was associated with greater CCDR severity; Comparative peripheral biomarker context	The canine cohort was not PD-specific, and marked age differences may confound the plasma NOX4 association. The relationship between circulating and central NOX4 biology remains undefined, and cognition was not assessed in the separate MPTP-treated mouse comparator.

Evidence categories for the 10 studies constituting the core evidence base are based on the author-defined evidence categorization described in Section 2.4 and primarily reflect how directly NOX involvement was tested in relation to cognitive outcomes. Where an *in vivo* intervention was directed at NOX, genetic manipulation was distinguished from NOX-targeted pharmacological intervention, while complementary mechanistic evidence was considered separately when NOX dependence was demonstrated outside the cognition-tested *in vivo* paradigm. The categories are descriptive rather than ordinal and are not intended to constitute a formal ranking of overall evidence strength or study quality. Direct genetic evidence denotes *in vivo* genetic manipulation of a NOX isoform or component with cognitive testing. NOX-targeted pharmacological intervention denotes *in vivo* administration of an agent employed explicitly to inhibit NOX with cognitive testing. This category reflects the intervention strategy rather than target-specific mediation and does not imply isoform, cell-type, or target specificity or establish that the cognitive effect was mediated specifically or exclusively through NOX inhibition. Partial evidence denotes within-study links between cognitive outcomes and NOX-related findings when NOX-dependent cognitive causality was not directly tested *in vivo* and NOX dependence was not established in complementary mechanistic experiments. Supportive evidence comprises isolated NOX-component findings without direct functional validation or sufficient convergent support. Supportive discordant evidence denotes findings in which the NOX-related measure did not show a consistent relationship with cognitive status across the comparisons examined. Complementary pathway-level evidence denotes studies in which cognition-related pathway components were examined *in vivo* and NOX dependence was demonstrated in complementary mechanistic experiments without direct *in vivo* NOX manipulation during cognitive assessment. This category describes a distinct form of mechanistic support and does not imply stronger *in vivo* NOX-dependent cognitive causality than partial evidence. The human postmortem and canine biomarker studies were retained as contextual studies and were not assigned the evidence categories used for the 10 core studies. For the 10 core studies, randomization and blinded outcome assessment were recorded only when explicitly reported in the article or supplementary materials. Absence of reporting was recorded as "not reported" and was not interpreted as evidence that the procedure was not performed. Reference numbers correspond to the main manuscript.

Abbreviations: AD, Alzheimer's disease; M, male; F, female; 6-OHDA, 6-hydroxydopamine; AAV, adeno-associated virus; CCDR, canine cognitive dysfunction rating; DFO, iron chelator; DG, dentate gyrus; DSP-4, N-(2-chloroethyl)-N-ethyl-2-bromobenzylamine hydrochloride; LPS, lipopolysaccharide; Mac1, macrophage-1 antigen; MCI, mild cognitive impairment; MPTP, 1-methyl-4-phenyl-1,2,3,6-tetrahydropyridine; MWM, Morris water maze; NLRP3, NLR family pyrin domain containing 3; NOR, novel object recognition; NOX, NADPH oxidase; P+M, paraquat plus maneb; p-p47phox, phosphorylated p47phox; PD, Parkinson's disease; prePD, presymptomatic PD; SCI, severe cognitive impairment; VD_3, vitamin D_3.
