# Supplementary material for: NADPH Oxidase-Related Oxidative Stress in Parkinson’s Disease-Associated Cognitive Impairment: An Evidence-Focused Review
Source: Antioxidants (Basel). 2026 Oct 21;15(10):1367. doi: 10.3390/antiox15101367 (PMC13701224; doi:10.3390/antiox15101367)
Supplement: Supplementary file 1 [file antioxidants-15-01367-s001.zip › Revised_Table_S2_References_Updated_2026-10-21.pdf]

Supplementary Table S2. Reports excluded from study-level synthesis and primary reasons for exclusion (n = 46)

The 46 excluded reports are grouped by one primary exclusion reason. To keep the report-level audit readable, the table reports first author/year, article title, journal, and a concise study-specific rationale. Full author lists and identifiers are omitted. Review articles excluded before report-level relevance assessment are not listed here.

Study	Article title	Journal	Primary exclusion rationale
**Outside the PD/PD-relevant scope, including false-positive retrievals (n = 25)**			
Albrecht-Goepfert et al., 1998 [36]	Modulation of the production of reactive oxygen species by pre-activated neutrophils by aminoadamantane derivatives	Biochemical Pharmacology	Pre-activated neutrophil ROS/NADPH oxidase study using aminoadamantane derivatives; no PD/PD-relevant model or cognitive outcome.
Liu et al., 2010 [61]	Apocynin attenuate spatial learning deficits and oxidative responses to intermittent hypoxia	Sleep Medicine	Intermittent-hypoxia/sleep-disordered-breathing model; cognition and NOX-related outcomes were assessed, but the disease context was outside PD/PD-relevant scope.
Lull et al., 2011 [64]	Chronic Apocynin Treatment Attenuates Beta Amyloid Plaque Size and Microglial Number in hAPP(751)SL Mice	PLoS ONE	Alzheimer-type hAPP transgenic model rather than PD or a PD-relevant cognitive model.
Ding et al., 2014 [43]	Elevated dopamine induces minimal hepatic encephalopathy by activation of astrocytic NADPH oxidase and astrocytic protein tyrosine nitration	International Journal of Biochemistry & Cell Biology	Minimal hepatic encephalopathy model. Cognitive impairment and astrocytic NADPH oxidase were examined, but the disease context was outside the predefined PD/PD-relevant scope.
Dimitrov et al., 2014 [42]	Anxiety- and depression-like behavior and impaired neurogenesis evoked by peripheral neuropathy persist following resolution of prolonged tactile hypersensitivity	Journal of Neuroscience	Peripheral neuropathic-pain model with Y-maze/NOR-related behavioral assessment; not a PD/PD-relevant model.
Modi et al., 2015 [67]	Cinnamon and Its Metabolite Sodium Benzoate Attenuate the Activation of p21rac and Protect Memory and Learning in an Animal Model of Alzheimer's Disease	PLoS ONE	Alzheimer's disease model rather than PD/PD-associated cognitive impairment.
Parajuli et al., 2015 [69]	CCL11 Enhances Excitotoxic Neuronal Death by Producing Reactive Oxygen Species in Microglia	GLIA	Excitotoxic/inflammatory neurotoxicity study without a PD or PD-relevant cognitive disease model.
Xu et al., 2016 [78]	Prevention of Hippocampal Neuronal Damage and Cognitive Function Deficits in Vascular Dementia by Dextromethorphan	Molecular Neurobiology	Vascular dementia model rather than PD/PD-associated cognitive impairment.
Cho et al., 2017 [40]	Neuronal Cell Death and Degeneration through Increased Nitroxidative Stress and Tau Phosphorylation in HIV-1 Transgenic Rats	PLoS ONE	HIV-1 transgenic neurodegeneration model rather than PD/PD-associated cognitive impairment.
Bhat et al., 2018 [37]	Angiotensin Receptor Blockade by Inhibiting Glial Activation Promotes Hippocampal Neurogenesis Via Activation of Wnt/β-Catenin Signaling in Hypertension	Molecular Neurobiology	Hypertension-related hippocampal neuroinflammation/neurogenesis study; not a PD/PD-relevant cognitive model.
Gupta and Kaur, 2018 [50]	Withania somnifera as a Potential Anxiolytic and Anti-inflammatory Candidate Against Systemic Lipopolysaccharide-Induced Neuroinflammation	Neuromolecular Medicine	Systemic LPS neuroinflammation/anxiety model rather than PD-associated cognitive impairment.

Study	Article title	Journal	Primary exclusion rationale
Kiely et al., 2018 [57]	Immunohistochemical and Molecular Investigations Show Alteration in the Inflammatory Profile of Multiple System Atrophy Brain	Journal of Neuropathology & Experimental Neurology	Multiple system atrophy neuropathology study rather than PD-associated cognitive impairment.
Adeluyi et al., 2019 [35]	Microglia morphology and proinflammatory signaling in the nucleus accumbens during nicotine withdrawal	Science Advances	Nicotine-withdrawal/addiction model; not PD/PD-associated cognitive impairment.
Weaver et al., 2019 [77]	McLeod syndrome: Five new pedigrees with novel mutations	Parkinsonism & Related Disorders	McLeod syndrome/neuroacanthocytosis case series; not a PD-associated cognition or NOX-focused PD study.
Hu et al., 2018 [54]	Renal proteomic analysis of RGC-32 knockout mice reveals the potential mechanism of RGC-32 in regulating cell cycle	American Journal of Translational Research	Renal proteomic study; PD appeared only as a pathway annotation and the study was outside the neurological inclusion scope.
Hu et al., 2022 [53]	Network Embedding Across Multiple Tissues and Data Modalities Elucidates the Context of Host Factors Important for COVID-19 Infection	Frontiers in Genetics	COVID-19/network-analysis study; not PD-associated cognitive impairment.
Hu et al., 2022 [52]	ZIF-8 nanoparticles induce neurobehavioral disorders through the regulation of ROS-mediated oxidative stress in zebrafish embryos	Chemosphere	Developmental neurotoxicity study in zebrafish embryos with ROS/NOX1-related changes; not a PD/PD-relevant model or cognition-specific study.
Magnusson et al., 2024 [65]	Porphyromonas gingivalis triggers microglia activation and neurodegenerative processes through NOX4	Frontiers in Cellular and Infection Microbiology	Periodontal-bacteria/microglial study framed toward Alzheimer-related neurodegeneration; not a PD/PD-relevant cognitive model.
Seligmann et al., 2024 [73]	Molecular Gene Expression Testing to Identify Alzheimer’s Disease with High Accuracy from Fingerstick Blood	Journal of Alzheimer's Disease	Alzheimer's disease blood-based gene-expression diagnostic study; not a PD/PD-relevant cognitive study.
Franklin et al., 2025 [45]	Effects of iron accumulation and its chelation on oxidative stress in intracortical implants	Acta Biomaterialia	Intracortical implant foreign-body injury study examining iron, NOX subunits, and oxidative stress; not a PD model or cognition study.
Gokce and Sekmen, 2025 [49]	Exploring the regulatory roles of AtGLR3.4 receptors in mitochondrial stress and ROS management in Arabidopsis	Plant Cell Reports	Arabidopsis plant mitochondrial-stress/ROS study; non-neurological and outside the PD/PD-relevant scope.
Kamihara et al., 2025 [56]	Deciphering the relationship between sarcopenia and aging: A combined text mining and gene expression analysis	Geriatrics & Gerontology International	Sarcopenia/aging bioinformatics study; not PD-associated cognitive impairment.
Punsawad et al., 2025 [70]	Low-Dose Paracetamol Treatment Protects Neuronal Oxidative Stress and Neuroinflammation in D-Galactose-Induced Accelerated Aging Model	Scientifica	D-galactose accelerated-aging model; not a PD/PD-relevant cognitive model.
Sinha et al., 2025 [74]	A New Differential Gene Expression Based Simulated Annealing for Solving Gene Selection Problem: A Case Study on Eosinophilic Esophagitis and Few Other Gastro-intestinal Diseases	Biochemical Genetics	Bioinformatics/gene-selection study focused on gastrointestinal diseases; unrelated to PD-associated cognition or NOX mechanisms.
Qin et al., 2026 [71]	NOX4 promotes cardiomyocyte ferroptosis-related injury in septic cardiomyopathy in associated with mitochondrial dysfunction and impaired NRF2 nuclear translocation	International Journal of Biological Macromolecules	Septic cardiomyopathy/cardiomyocyte ferroptosis study; non-CNS and outside the PD/PD-relevant scope.

Study	Article title	Journal	Primary exclusion rationale
**No eligible cognitive outcome or cognition-defined status (n = 12)**			
Jin et al., 2007 [55]	Prostaglandin E2 receptor subtype 2 (EP2) regulates microglial activation and associated neurotoxicity induced by aggregated alpha-synuclein	Journal of Neuroinflammation	PD/α-synuclein-relevant microglial study, but no eligible cognitive outcome or cognition-defined status was assessed.
Hemmati-Dinarvand et al., 2017 [51]	Dysregulation of serum NADPH oxidase1 and ferritin levels provides insights into diagnosis of Parkinson's disease	Clinical Biochemistry	Human PD NOX1 biomarker study without cognitive testing or cognition-defined subgroup analysis.
Chen et al., 2018 [39]	Therapeutic effects of honokiol on motor impairment in hemiparkinsonian mice are associated with reversing neurodegeneration and targeting PPARγ regulation	Biomedicine & Pharmacotherapy	PD model focused on motor impairment and neurodegeneration; no eligible cognitive outcome.
Lee et al., 2019 [59]	The phosphodiesterase 10 inhibitor papaverine exerts anti-inflammatory and neuroprotective effects via the PKA signaling pathway in neuroinflammation and Parkinson's disease mouse models	Journal of Neuroinflammation	MPTP PD mouse model with locomotor testing (including rotarod/pole test), but no eligible learning or memory outcome.
Song et al., 2019 [75]	Loss of Brain Norepinephrine Elicits Neuroinflammation-Mediated Oxidative Injury and Selective Caudo-Rostral Neurodegeneration	Molecular Neurobiology	Noradrenergic/PD-relevant neuroinflammation study without an eligible cognitive outcome in the reported experiment.
Garofalo et al., 2020 [48]	Alzheimer's, Parkinson's Disease and Amyotrophic Lateral Sclerosis Gene Expression Patterns Divergence Reveals Different Grade of RNA Metabolism Involvement	International Journal of Molecular Sciences	Human PD transcriptomic/gene-expression study without cognitive testing or cognition-defined PD subgroup analysis.
Loffredo et al., 2020 [63]	Oxidative Stress and Gut-Derived Lipopolysaccharides in Neurodegenerative Disease: Role of NOX2	Oxidative Medicine and Cellular Longevity	NOX2/redox study relevant to neurodegenerative disease, but no eligible cognition-linked PD outcome was assessed.
de Lima et al., 2022 [41]	Effects of vitamin D (VD3) supplementation on the brain mitochondrial function of male rats, in the 6-OHDA-induced model of Parkinson's disease	Neurochemistry International	6-OHDA PD rat study focused on mitochondrial function and dopaminergic/mitochondrial markers; no eligible learning or memory assessment.
Nascimento et al., 2022 [68]	Oxidative Stress Inhibition Via Apocynin Prevents Medullary Respiratory Neurodegeneration and Respiratory Pattern Dysfunction in a 6-Hydroxydopamine Animal Model of Parkinson's Disease	Neuroscience	PD model with NOX inhibition focused on respiratory and motor outcomes; no cognitive outcome.
Feng et al., 2024 [44]	Cycloastragenol reduces microglial NLRP3 inflammasome activation in Parkinson's disease models by promoting autophagy and reducing Scrib-driven ROS	Phytomedicine	PD neuroinflammation study with ROS-related mechanisms, but no eligible cognitive outcome.
Rodriguez-Perez et al., 2020 [72]	Angiotensin type 2 receptors: Role in aging and neuroinflammation in the substantia nigra	Brain, Behavior, and Immunity	Nigral aging/neuroinflammation study with NADPH-oxidase-related signaling, but no cognitive outcome.
Wang et al., 2014 [76]	Substance P exacerbates dopaminergic neurodegeneration through neurokinin-1 receptor-independent activation of microglial NADPH oxidase	Journal of Neuroscience	PD-relevant dopaminergic neurodegeneration/NOX study without cognitive assessment.

Study	Article title	Journal	Primary exclusion rationale
**In vitro/cell-based evidence only (n = 5)**			
Lo et al., 2008 [62]	Neuronal effects of 4-t-Butylcatechol: A model for catechol-containing antioxidants	Toxicology and Applied Pharmacology	Cell-based neuronal oxidative-stress study without an in vivo or clinical cognitive outcome.
Yap et al., 2010 [79]	Temporal transcriptomic profiling reveals cellular targets that govern survival in HOCl-mediated neuronal apoptosis	Life Sciences	In vitro neuronal apoptosis/transcriptomic study without an in vivo or clinical cognitive outcome.
Gan et al., 2015 [47]	Anti-inflammatory effects of glaucocalyxin B in microglia cells	Journal of Pharmacological Sciences	Microglial cell study only; no in vivo or clinical cognitive outcome.
Gallardo-Fernández et al., 2019 [46]	Hydroxytyrosol Decreases LPS- and α-Synuclein-Induced Microglial Activation In Vitro	Antioxidants	In vitro microglial activation study only; no in vivo or clinical cognitive outcome.
Manokaran et al., 2018 [66]	Effect of simvastatin on neuroinflammation in microglial cells via mitogen-activated protein kinase and nuclear factor κB pathways	Pharmacognosy Magazine	LPS-activated microglial cell study without an in vivo PD cognitive outcome.
**No eligible NOX-related assessment (n = 3)**			
Kim et al., 2015 [58]	α-Asarone attenuates microglia-mediated neuroinflammation by inhibiting NF-κB activation and mitigates MPTP-induced behavioral deficits in a mouse model of Parkinson's disease	Neuropharmacology	PD model with Y-maze testing, but no eligible NOX/NADPH oxidase assessment or manipulation was identified.
Chen et al., 2020 [38]	Protective role of cinnabar and realgar in Hua-Feng-Dan against LPS plus rotenone-induced neurotoxicity and disturbance of gut microbiota in rats	Journal of Ethnopharmacology	PD/parkinsonian model with Y-maze testing, but no eligible NOX/NADPH oxidase assessment or manipulation was identified.
Li et al., 2026 [60]	Plasma proteome mediates the associations between air pollution exposure and disease risk	Nature Communications	Air-pollution/proteomics study in which NOx denotes nitrogen oxides rather than NADPH oxidase; no eligible NOX-related assessment.
**Non-independent publication (n = 1)**			
Hou et al., 2025	Corrigendum to 'Inhibition of NADPH oxidase by apocynin prevents learning and memory deficits in a mouse Parkinson's disease model'	Redox Biology	Corrigendum to an already included original study; not counted as an independent report.

Note. Each report was assigned one primary exclusion reason for transparency of study selection. The categories summarize why a report did not enter the final set of 12 studies retained for study-level synthesis and are not judgments of overall study quality.
